# New species and new records of Mydidae from the Afrotropical and Oriental regions (Insecta, Diptera, Asiloidea)

**DOI:** 10.3897/zookeys.64.464

**Published:** 2010-10-22

**Authors:** Torsten Dikow

**Affiliations:** Biodiversity Synthesis Center, Field Museum of Natural History, 1400 S. Lake Shore Drive, Chicago, IL 60605, USA

**Keywords:** Mydidae, Leptomydinae, Syllegomydinae, Afrotropical, Oriental, biodiversity hotspots

## Abstract

New Mydidae species are described from the Afrotropical and Oriental regions including the first records of this family from several countries in eastern Africa (Kenya, Tanzania, and Uganda) and Mauritania in western Africa as well as Nepal and Thailand in Asia. The new species are, Leptomydinae: Leptomydas notos **sp. n.** (south-western India), Leptomydas rapti **sp. n.** (south-central Nepal), Leptomydas tigris **sp. n.** (north-central Thailand); Syllegomydinae: Mydaselpidini: Mydaselpis ngurumani **sp. n.** (south-eastern Kenya, north-eastern Tanzania), Vespiodes phaios **sp. n.** (south-eastern Kenya); Syllegomydinae: Syllegomydini: Syllegomydas (Notobates) astrictus **sp. n.** (Kenya), Syllegomydas (Notobates) heothinos **sp. n.** (Kenya and Uganda), Syllegomydas (Syllegomydas) elachys **sp. n.** (northern Zimbabwe). Syllegomydas (Syllegomydas) proximus Séguy, 1928 is recorded from western Mauritania and re-described. Syllegomydas (Notobates) dispar (Loew, 1852), which was previously listed as *incertae sedis* in the Afrotropical Diptera catalogue, is re-described and illustrated based on examination of the type specimens and several additional specimens from Mozambique. Cephalocera annulata Brunetti, 1912 and Syllegomydas bucciferus Séguy, 1928, described from north-eastern India and previously unplaced in the Oriental Diptera catalogue, are newly combined with Leptomydas Gerstaecker, 1868 and together with Leptomydas indianus Brunetti, 1912, also from north-eastern India, placed in Leptomydinae. Comments on the possible synonymy of the genera of Mydaselpidini are made. Illustrations and photographs are provided to support the descriptions and future identification. A provisional dichotomous key to Mydidae genera occurring in eastern Africa (Kenya, Malawi, Mozambique, Somalia, Tanzania, Uganda) and the Oriental Region is provided. Distribution, occurrence in biodiversity hotspots and high-biodiversity wilderness areas, and seasonal incidence are discussed for all species.

## Introduction

Mydidae is with 463 species in 66 genera one of the less speciose families of Asiloidea (Diptera: Brachycera). The taxon is distributed in warmer climates throughout the world, but shows the highest species diversity in southern Africa. Although regional faunas are in several instances fairly well known, e.g., North America, Australia, or Chile, there are still many areas where new species will be found and need to be made scientifically known in the future. The aim of this publication is to fill some of the distributional gaps world-wide and report for the first time Mydidae from eastern Africa (Kenya, Tanzania, and Uganda) and Mauritania in the Afrotropical Region and Nepal and Thailand in the Oriental Region. In addition, apparently undescribed species are described from India and Zimbabwe and the placement of several species from the Afrotropical and Oriental regions previously considered as *incertae sedis* is addressed.

## Materials and methods

Morphological terminology and abbreviations for setae follow McAlpine (1981) and Dikow (2009) except for the term ‘aedeagal epimere’, which is used as described by Hesse (1969). Abdominal tergites are abbreviated in the descriptions with ‘T,’ and sternites are abbreviated with ‘S.’ Other generalised terms refer to the *Torre-Bueno Glossary of Entomology* (Nichols 1989). The adjective pruinose is used here for short, fine cuticular microtrichia that densely cover certain body parts and reflect light in a different way to that of bare cuticle. The species descriptions are based on composites of all specimens and not exclusively on the holotype and are compiled from a character matrix of 144 features assembled with Lucid Builder (version 3.5) and eventually exported as natural language descriptions. When available, species are fully described in the male sex while females are only described with those features that differ (except for characters relating to the terminalia/genitalia). The descriptions and re-descriptions are very detailed on purpose in order to allow proper identification in the future as it is likely that additional species will be found within the areas covered in this publication.

The female genitalia and male terminalia were first excised and macerated in 10% potassium hydroxide (KOH) at 55°C followed by rinsing in distilled H_2_O. They were temporarily stored in 75% ethanol for examination and illustration and eventually sealed in polyethylene genitalia vials containing 100% glycerine and attached to the specimen’s pin. Morphological features were illustrated using a 10×10 ocular grid on a Olympus SZ60 stereo microscope and later digitally redrawn using Adobe Illustrator® software. The vestiture on male terminalia is not shown. Wing length was measured from the tegula to the distal tip of the wing. Photographs of pinned specimens were taken with a Olympus E-30 digital SLR, a 50 mm macro lens (equivalent of 100 mm focal length in 35 mm photography), and a 25 mm extension tube. The specimens were illuminated by a LED ring-light fitted with a dome for even and soft light.

In recording data for type specimens as well as non-type specimens, information is given (where available) in a standard manner, i.e., locality, geographic co-ordinates, elevation, date of collection (month indicated in lower case Roman numerals where hyphens indicate missing entries for day, month, year), habitat information, collector, and depository. Female (♀) and male (♂) symbols indicate the sex while a question mark (?) refers to specimens of indeterminable sex (i.e., with broken or missing abdomen). Each specimen is listed with a unique AAM specimen number that will allow the re-investigation as well as provide a unique identifier (LSID http://lsids.sourceforge.net/) in databases like GBIF (http://www.gbif.org/) in the future. AAM is an abbreviation for ‘Apioceridae Asilidae Mydidae’ and identifies a record in the specimen database used by the author in this format: AAM-000000. The distribution is illustrated in distribution maps with all localities plotted, for which coordinates were available, and the type locality is plotted with an open symbol. The electronic shape-files of the Biodiversity Hotspots and High-biodiversity Wilderness Areas were obtained from Conservation International (2005).

Institutions providing specimens are listed below, together with the abbreviations used in the text when citing depositories, and the people who kindly assisted: AMGS - Albany Museum, Grahamstown, Eastern Cape, South Africa (A. Kirk-Spriggs, S. Gess); BMNH - The Natural History Museum, London, UK (E. McAlister); CAS - California Academy of Sciences, San Francisco, California, USA (C. Griswold); CNC - Canadian National Collection of Insects, Arachnids and Nematodes, Ottawa, Ontario, Canada (J. Skevington); LACM - Natural History Museum of Los Angeles County, Los Angeles, California, USA (B. Brown); MNHN - Museum national d’Histoire naturelle, Paris, France (C. Daugeron, E. Delfosse); NMSA - Natal Museum, Pietermaritzburg, KwaZulu-Natal, South Africa (J. Londt, G. Davies); NMKE - National Museums of Kenya, Nairobi, Kenya (R. Copeland); TMSA - National Museum of Natural History (Transvaal Museum), Pretoria, Gauteng, South Africa (R. Lyle); ZMHB - Museum für Naturkunde, Berlin, Germany (J. Ziegler, J. Pohl); ZSMC - Zoologische Staatssammlung, München, Bayern, Germany (M. Kotrba).

## Taxonomy

This publication deals with a diverse Mydidae fauna, both in terms of geography as species from the Afrotropical and Oriental regions are dealt with as well as in terms of genera as 4 genera from 2 subfamily taxa are included. In order to allow the identification of the Mydidae genera occurring in India, Kenya, Nepal, Tanzania, Thailand, and Uganda, from which the new species are being described, a provisional generic key is provided below. This key excludes Nemomydas Curran, 1934 as it is only known from Taiwan and the Yaeyama Islands of Japan in the far eastern Oriental Region, but comments on this genus are made in the Discussion. For further details about the delimitation of other genera see below and the Discussion. An updated, illustrated identification key to all 11 currently recognized subfamily taxa, which is based on the key by Papavero and Wilcox (1974), can be accessed on this web-site: http://www.mydidae.tdvia.de/online_keys.

**Table:** 

1	Specimens from the Oriental Region	5
–	Specimens from eastern Africa (Kenya, Malawi, Mozambique, Somalia, Tanzania, Uganda)	2
2	Proboscis short or very short, at most ½ the length of oral cavity; metathoracic femora cylindrical, only slightly wider than prothoracic and mesothoracic femora (e.g., Fig. 41); anatergal setae present; supero-posterior anepisternum short or long setose Syllegomydas Becker, 1906
–	Proboscis long, reaching or extending beyond fronto-clypeal suture; metathoracic femora distinctly clubbed, much wider than prothoracic and mesothoracic femora; anatergal setae absent; supero-posterior anepisternum asetose	3
3	Alula reduced, nearly straight wing margin (Fig. 43); interocular distance on vertex as wide as at ventral eye margin; ♀ ovipositor with acanthophorite spurs	Mydaselpis ngurumani sp. n.
–	Alula well-developed (e.g., Fig. 42); interocular distance on vertex larger than at ventral eye margin; ♀ ovipositor without acanthophorite spurs	4
4	♂ aedeagal epimere absent	Mydaselpis Bezzi, 1924
–	♂ aedeagal epimere present, distally simple and evenly rounded	Vespiodes Hesse, 1969
–	♂ aedeagal epimere present, distally bifurcate	Afromydas Bequaert, 1961
5	m_3_+CuA_1_ do not terminate together in C (Fig. 42); metathoracic tibiae without ventral keel; mystax more or less evenly distributed over facial gibbosity, mystax can be sparse medially; ♀ ovipositor with acanthophorite spurs	Leptomydas Gerstaecker, 1868
–	m_3_+CuA_1_ terminate together in C, reaching posterior wing margin; metathoracic tibiae with ventral keel proximally; mystax formed by distinct crest of densely arranged macrosetae along fronto-clypeal suture, mystax not developed on dorso-median facial gibbosity; ♀ ovipositor without acanthophorite spurs	(Cacatuopyginae) 6
6	Alula well-developed, medially reaching scutellum; predominantly black species; scutum either apruinose or entirely black pruinose	Cacatuopyga Papavero & Wilcox, 1974
–	Alula reduced to small lobe; predominantly brown species; scutum at least laterally grey pruinose	Charimydas Bowden, 1984

### Leptomydinae

The Leptomydinae comprises 47 valid species in 6 genera primarily distributed in the Northern Hemisphere with Hessemydas Kondratieff, Carr and Irwin, 2005 from Madagascar and Plyomydas Wilcox and Papavero, 1971 from Peru being the only representatives in the Southern Hemisphere.

#### Leptomydas Gerstaecker, 1868

Leptomydas has currently 12 valid species distributed in the southern Palaearctic and north-western Oriental regions.

##### Leptomydas notos sp. n.

urn:lsid:zoobank.org:act:26BD416E-6E07-44FB-9450-9AACE98E9021

Figs 1–3 30 42 46 

###### Etymology:

*notos* Greek adjective = south. Referring to the apparent distribution in southern India in the state of Tamil Nadu.

###### Diagnosis:

The species is distinguished from congeners by the short proboscis that does not project beyond the fronto-clypeal suture, the short setose brown to bluish-black abdominal tergites with light yellow posterior margins, features of the male terminalia, and its apparent distribution in south-western India.

###### Description Male:

Head: brown, in general white pruinose; width distinctly greater than thorax, interocular distance on vertex larger than at ventral eye margin, vertex between compound eyes slightly depressed, parafacial area less than ½ the width of central facial gibbosity; facial gibbosity distinct, well-developed and discernible in lateral view; mystax white, covering entire facial gibbosity; frons predominantly apruinose, vertex apruinose, postgenae apruinose; setation: vertex white, frons white, ocp setae white, pocl setae white; ocellar triangle apruinose; proboscis brown, short, about ½ length of oral cavity; labella large, much wider than prementum, as long as prementum, unsclerotised laterally; maxillary palpi cylindrical, brown, about ⅓ the length of proboscis.

Antenna: brown, scape and pedicel white setose dorsally and ventrally; postpedicel cylindrical in proximal ½, symmetrically bulbous in distal ½, ≥ 6.0 times as long as combined length of scape and pedicel; apical ‚seta-like‘ sensory element situated apically in cavity on postpedicel.

Thorax: brown, scutum predominantly grey pruinose, pleura predominantly apruinose; scutum medially bluish-black, laterally brown, surface entirely smooth, predominantly grey pruinose, only broad sublateral stripes and narrow paramedial stripes (not reaching posterior margin) apruinose, scutal setation comprised of distinct rows of long dorsocentral setae and lateral scutal setae; dc setae pre- and postsuturally white, acr setae absent, lateral scutal setae white, npl, spal, and pal setae absent; postpronotal lobe light brown, grey pruinose; proepisternum, lateral postpronotum, and postpronotal lobes long white setose; scutellum grey pruinose proximally, apruinose distally, asetose, apical scutellar setae absent; mesopostnotum, anatergite, and katatergite grey pruinose, mesopostnotum asetose, anatergite asetose, katatergite long white setose; katatergite ± flat; anterior anepisternum asetose, supero-posterior anepisternum asetose; posterior anepimeron densely long white setose, katepimeron asetose; metepimeron ± flat, same colour as T1, grey pruinose, long white setose; metepisternum grey pruinose, asetose.

Leg: brown or light brown, setation predominantly white; all coxae apruinose, white and brown setose; met trochanter setose medially; femora brown or light brown, met femora evenly clubbed in distal ¾, in distal ½ macrosetose, 1 antero-ventral and 1 postero-ventral row of macrosetae; all tibiae laterally arched, met tibia cylindrical, ventral keel absent; pro and mes tarsomere 1 about as long as individual tarsomeres 2, 3, or 4, met tarsomere 1 as long as combined length of tarsomeres 2–3; pulvilli well-developed, as long as well-developed claws, and as wide as base of claws; empodium absent.

Wing: length = 8.9–9.9 mm; slightly brown stained throughout, veins light brown, microtrichia absent; cells r_1_, r_4_, r_5_, m_3_, + cu*p* closed; C terminates at junction with R_1_; R_4_ terminates in R_1_; R_5_ terminates in R_1_; stump vein (R_3_) at base of R_4_ present, short not reaching R_2_; R_4_ and R_5_ widest apart medially; r-m indistinct, R_4+5_ and M_1_ fused; M_1_ straight at r-m (not curving anteriorly), M_1_ (or M_1_+M_2_) terminates in R_1_; CuA_1_ and CuA_2_ split proximally to m-cu (cell m_3_ narrow proximally); M_3_+CuA_1_ do not terminate together in C; A_1_ undulating, cell a_1_ wide, A_1_ and wing margin further apart proximally than distally, alula well-developed; halter brown.

Abdomen: brown to bluish-black; setation comprised of scattered white and brown setae, surface entirely smooth; T1–7 brown, yellow posterior margins; T1 and anterior ¼ of T2 long white setose, remaining T2 and T3 brown and white setose; T predominantly apruinose; S1–7 brown, yellow posterior margins; S1 asetose, S2–3 sparsely brown setose; S predominantly apruinose; T2–4 parallel-sided and not constricted waist-like; bullae on T2 brown, oval, surface entirely smooth, T2 surface anterior to bullae smooth.

Male terminalia: T1–7 well-developed, entirely sclerotised, T8 postero-medially weakly sclerotised, with anterior transverse sclerotised bridge connecting lateral sclerites; T7–8 anteriorly with 2 lateral apodemes; S6 regular, without any special setation postero-medially, S8 well-developed and simple, not fused to T8 dorso-laterally, entire (undivided) ventro-medially; epandrium formed by single sclerite (fused medially ± entirely), blunt, evenly rounded; subepandrial sclerite without lateral or median protuberances; hypandrium slightly concave, divided ventro-medially by unsclerotised area into 2 separate sclerotised halves, entirely fused with gonocoxite, forming a gonocoxite-hypandrial complex; gonocoxites dorso-ventrally flattened (same height throughout, expanded laterally and medially), with median protuberance, gonocoxal apodeme absent; 1 functional aedeagal prong; aedeagal epimere absent; lateral ejaculatory processes absent; ejaculatory apodeme formed by single dorso-ventrally oriented plate; ventro-median margin of dorsal aedeagal sheath heavily sclerotised (appearing entirely closed); dorsal aedeagal sheath long, sperm sac entirely covered; sperm sac appearing ± heavily sclerotised.

###### Description Female:

unknown.

**Figures 1–9.**
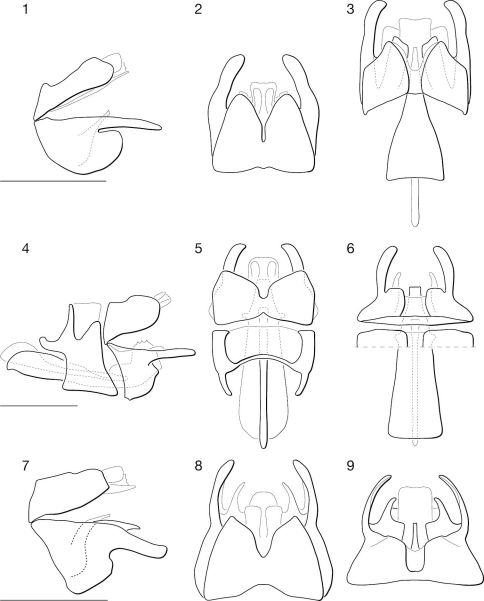
♂ terminalia of Leptomydas species. **1–3.** Leptomydas notos sp. n. **1** lateral **2** dorsal **3** ventral. **4–6.** Leptomydas rapti sp. n. **4** lateral **5** dorsal **6** ventral. **7–9.** Leptomydas tigris sp. n. **7** lateral **8** dorsal **9** ventral. Scale lines = 1 mm.

###### Material examined:

**India:** Tamil Nadu: 7 ♂ Coimbatore, 10°59'33"N; 076°57'41"E, 21.v.1946, on sand dry river bed, P. Nathan (AAM-000776 1 ♂ holotype, AAM-000770–AAM-000775 paratypes, AMNH).

###### Type locality and distribution:

Coimbatore (10°59'33"N; 076°57'41"E), India (Fig. 46). Biodiversity hotspot/high-biodiversity wilderness area: Western Ghats and Sri Lanka/-.

##### Leptomydas rapti sp. n.

urn:lsid:zoobank.org:act:E4EEC8E2-C144-4884-89EF-CC4C369CAA2A

Figs 4–6 31 46 

###### Etymology:

The specific epithet refers to the Rapti Valley, Nepal in which the type locality is situated and is a noun in apposition.

###### Diagnosis:

The species is distinguished from congeners by the entirely light brown stained wings, the long brown setae on the posterior abdominal sternites, the fusion of abdominal sternite 8 with tergite 8, and its apparent distribution in south-central Nepal.

###### Description Male:

Head: brown, in general grey pruinose; width distinctly greater than thorax, interocular distance on vertex larger than at ventral eye margin, vertex between compound eyes ± horizontally straight, medially only slightly below dorsal eye margin, parafacial area less than ½ the width of central facial gibbosity; facial gibbosity distinct, well-developed and discernible in lateral view; mystax white, covering only lateral facial gibbosity (asetose medially); frons medially apruinose, laterally grey pruinose, vertex predominantly apruinose, only lateral margins grey pruinose, postgenae apruinose; setation: vertex white, frons white, ocp setae white, pocl setae white; ocellar triangle apruinose; proboscis brown, long, projecting beyond fronto-clypeal suture; labella large, much wider than prementum, as long as prementum, unsclerotised laterally; maxillary palpi cylindrical, brown, shorter than length of pedicel.

Antenna: brown, scape and pedicel white setose dorsally and ventrally; postpedicel cylindrical in proximal ½, symmetrically bulbous in distal ½, ≥ 6.0 times as long as combined length of scape and pedicel; apical ‚seta-like‘ sensory element situated apically in cavity on postpedicel.

Thorax: brown, scutum predominantly grey pruinose, pleura predominantly apruinose; scutum medially bluish-black, laterally brown, surface entirely smooth, predominantly grey pruinose, only broad sublateral stripes and narrow paramedial stripes (not reaching posterior margin) apruinose, scutal setation comprised of distinct rows of long dorsocentral setae and lateral scutal setae; dc setae pre- and postsuturally white, acr setae absent, lateral scutal setae white, npl, spal, and pal setae absent; postpronotal lobe light brown, grey pruinose; proepisternum, lateral postpronotum, and postpronotal lobes long brown setose or long white setose; scutellum grey pruinose proximally, apruinose distally, asetose, apical scutellar setae absent; mesopostnotum, anatergite, and katatergite grey pruinose, mesopostnotum asetose, anatergite asetose, katatergite long white setose; katatergite ± flat; anterior anepisternum asetose, supero-posterior anepisternum asetose; posterior anepimeron long white setose, katepimeron asetose; metepimeron ± flat, same colour as T1, grey pruinose, long white setose; metepisternum grey pruinose, asetose.

Leg: brown, setation predominantly white; all coxae apruinose, long white setose; met trochanter setose medially; femora brown, met femora evenly clubbed in distal ¾, in distal ½ macrosetose, 1 antero-ventral and 1 postero-ventral row of macrosetae; all tibiae laterally arched, met tibia cylindrical, ventral keel absent; pro and mes tarsomere 1 about as long as individual tarsomeres 2, 3, or 4, met tarsomere 1 as long as combined length of tarsomeres 2–3; pulvilli well-developed, as long as well-developed claws, and as wide as base of claws; empodium absent.

Wing: length = 9.0–9.3 mm; slightly brown stained throughout, veins light brown, microtrichia absent; cells r_1_, r_4_, r_5_, m_3_, + cu*p* closed except r_5_ open; C terminates at junction with M_1_ (or M_1_+M_2_); R_4_ terminates in R_1_; R_5_ terminates in R_1_; stump vein (R_3_) at base of R_4_ present, short not reaching R2; R_4_ and R_5_ widest apart medially; r-m distinct, R_4_+_5_ and M_1_ apart, connected by crossvein or indistinct, R_4_+_5_ and M_1_ fused; M_1_ curves anteriorly at r-m, M_1_ (or M_1_+M_2_) terminates in C; CuA_1_ and CuA_2_ split proximally to m-cu (cell m_3_ narrow proximally); m_3_+CuA_1_ do not terminate together in C; A_1_ undulating, cell A_1_ wide, A_1_ and wing margin further apart proximally than distally, alula well-developed; halter brown.

Abdomen: brown to bluish-black; setation comprised of scattered white setae, surface entirely smooth; T1–7 brown, yellow posterior margins; T1 and anterior ½ of T2 long white setose, remaining T2 and T3 short white setose; T predominantly apruinose; S1–7 brown, yellow posterior margins; S1 asetose, S2 long white setose, S3 short white setose; S predominantly apruinose; T2–4 parallel-sided and not constricted waist-like; bullae on T2 brown, transversely elongate, surface entirely smooth, T2 surface anterior to bullae smooth.

**Male terminalia:** T1–7 well-developed, entirely sclerotised, T8 postero-medially weakly sclerotised, with anterior transverse sclerotised bridge connecting lateral sclerites; T7–8 anteriorly with 2 lateral apodemes; S6 regular, without any special setation postero-medially, S8 medially divided into two halves, fused to T8 dorso-laterally, divided entirely ventro-medially; epandrium formed by single sclerite (fused medially ± entirely), blunt, evenly rounded; subepandrial sclerite without lateral or median protuberances; hypandrium slightly concave, partially divided ventro-medially, anteriorly with heavily sclerotised transverse bridge, entirely fused with gonocoxite, forming a gonocoxite-hypandrial complex; gonocoxites dorso-ventrally flattened (same height throughout, expanded laterally and medially), with median protuberance, gonocoxal apodeme absent; 1 functional aedeagal prong; aedeagal epimere absent; lateral ejaculatory processes absent; ejaculatory apodeme formed by single dorso-ventrally oriented plate; ventro-median margin of dorsal aedeagal sheath heavily sclerotised (appearing entirely closed); dorsal aedeagal sheath short, sperm sac entirely free; sperm sac appearing ± heavily sclerotised.

###### Description Female:

unknown.

###### Material examined:

**Nepal:** Bagmati: 1 ♂ Megouli (= Megauli), Rapti Valley, 27°35'00"N; 084°14'00"E, 29.iii.–4.iv.1962, 300 m, G. Ebert H. Falkner (AAM-000113 paratype, ZSMC); 2 ♂ Jhawani, Rapti Valley, 27°35'00"N; 084°31'00"E, 200 m, 16.iv.1967, Dierl Forster Schacht (AAM-000115 1 ♂ holotype, AAM-000114 paratype, ZSMC).

###### Type locality and distribution:

Jhawani (27°35'00"N; 084°31'00"E), Nepal (Fig. 46). Biodiversity hotspot/high-biodiversity wilderness area: Himalaya/-.

###### Remarks:

The particular shape of the tip of the aedeagus, which is not tapered distally (Fig. 4) as in other members of Leptomydas, is generally indicative of a species of Eremomidas Semenov, 1896 as suggested by Richter and Ovtshinnikova (1996) and Richter (1997). However, the proboscis of this species is projecting well-beyond the fronto-clypeal suture, a feature considered to be specific to Leptomydas. The correct generic placement of this species needs to be established with a phylogenetic analysis of Mydidae genera that is currently in preparation by the author.

##### Leptomydas tigris sp. n.

urn:lsid:zoobank.org:act:4D8128BB-1F62-431A-8A7B-9F03D664075D

Figs 7–9 32 46 

###### Etymology:

*tigris* Latin noun = tiger. Referring to the TIGER project (Thailand Inventory Group for Entomological Research) that collected the sole holotype in its extensive malaise trap sampling throughout Thailand.

###### Diagnosis:

The species is distinguished from congeners by the yellow and brown abdominal tergites, features of the male terminalia, and its apparent distribution in Thailand.

###### Description Male:

Head: brown, in general grey pruinose; width distinctly greater than thorax, interocular distance on vertex larger than at ventral eye margin, vertex between compound eyes slightly depressed, parafacial area less than ½ the width of central facial gibbosity; facial gibbosity distinct, well-developed and discernible in lateral view; mystax white, covering only lateral facial gibbosity (asetose medially); frons medially apruinose, laterally grey pruinose, vertex medially apruinose, laterally grey pruinose, postgenae apruinose; setation: vertex white, frons white, ocp setae white, pocl setae white; ocellar triangle apruinose; proboscis brown, long, projecting well-beyond fronto-clypeal suture, reaching ¼ of length of postpedicel; labella large, much wider than prementum, as long as prementum, unsclerotised laterally; maxillary palpi cylindrical, brown, as long as pedicel.

Antenna: brown, scape and pedicel white setose dorsally and ventrally; postpedicel cylindrical in proximal ½, symmetrically bulbous in distal ½, ≥ 7.0 times as long as combined length of scape and pedicel; apical ‚seta-like‘ sensory element situated apically in cavity on postpedicel.

Thorax: brown, scutum predominantly grey pruinose, pleura predominantly apruinose; scutum medially bluish-black, laterally brown, surface entirely smooth, predominantly pruinose with a presutural apruinose stripe antero-laterally, median stripe and sublateral stripes sparsely grey pruinose, remaining parts densely grey pruinose, scutal setation comprised of distinct rows of long dorsocentral setae and lateral scutal setae; dc setae pre- and postsuturally white, acr setae absent, lateral scutal setae white, npl, spal, and pal setae absent; postpronotal lobe brown, grey pruinose; proepisternum, lateral postpronotum, and postpronotal lobes long white setose; scutellum grey pruinose proximally, apruinose distally, asetose, apical scutellar setae absent; mesopostnotum, anatergite, and katatergite grey pruinose, mesopostnotum asetose, anatergite asetose, katatergite long white setose; katatergite ± flat; anterior anepisternum asetose, supero-posterior anepisternum asetose; posterior anepimeron long white setose, katepimeron asetose; metepimeron ± flat, same colour as T1, grey pruinose, long white setose; metepisternum grey pruinose, asetose.

Leg: brown and yellow, setation black and white; all coxae grey pruinose, white setose; met trochanter setose medially; femora anteriorly brown, posteriorly yellow, met femur proximally yellow, met femora ± cylindrical only slightly wider than pro and mes femora, in distal ½ macrosetose, only a single antero-ventral macroseta distally; all tibiae laterally arched, met tibia cylindrical, ventral keel absent; pro and mes tarsomere 1 about as long as individual tarsomeres 2, 3, or 4, met tarsomere 1 as long as combined length of tarsomeres 2–3; pulvilli well-developed, as long as well-developed claws, and as wide as base of claws; empodium absent.

Wing: length = 11.9 mm; slightly brown stained throughout, veins brown, microtrichia absent; cells r_1_, r_4_, r_5_, m_3_, + cu*p* closed except r_5_ open; C terminates at junction with M_1_ (or M_1_+M_2_); R_4_ terminates in R_1_; R_5_ terminates in R_1_; stump vein (R_3_) at base of R_4_ present, short not reaching R_2_; R_4_ and R_5_ widest apart medially; r-m distinct, R_4+5_ and M_1_ apart, connected by crossvein; M_1_ curves anteriorly at r-m, M_1_ (or M_1_+M_2_) terminates in C; CuA_1_ and CuA_2_ split proximally to m-cu (cell m_3_ narrow proximally); M_3_+CuA_1_ do not terminate together in C; A_1_ undulating, cell a_1_ wide, A_1_ and wing margin further apart proximally than distally, alula well-developed; halter brown or light brown.

Abdomen: brown and yellow; setation comprised of scattered white and black setae, surface entirely smooth; T1 and T5–7 brown, narrow yellow posterior margins, T2–4 yellow, brown medially and laterally; T1–2 long white setose, T3 short white and black setose; T predominantly apruinose; S1 light brown, S2–5 yellow, brown anteriorly, S6–7 brown with yellow posterior margins; S1 asetose, S2 long white setose, S3 short white setose; S predominantly apruinose; T2–4 parallel-sided and not constricted waist-like; bullae on T2 brown, oval, surface entirely smooth, T2 surface anterior to bullae smooth.

Male terminalia: T1–7 well-developed, entirely sclerotised, T8 postero-medially weakly sclerotised, with anterior transverse sclerotised bridge connecting lateral sclerites; T7–8 anteriorly with 2 lateral apodemes; S6 regular, without any special setation postero-medially, S8 well-developed and simple, fused to T8 dorso-laterally, entire (undivided) ventro-medially; epandrium formed by single sclerite (fused medially ± entirely), pointed postero-medially; subepandrial sclerite without lateral or median protuberances; hypandrium slightly concave, partially divided ventro-medially, anteriorly with heavily sclerotised transverse bridge, entirely fused with gonocoxite, forming a gonocoxite-hypandrial complex; gonocoxites laterally compressed, slightly angled medially distally, with median protuberance, gonocoxal apodeme present, short (at most slightly extending hypopygium anteriorly); 1 functional aedeagal prong; aedeagal epimere absent; lateral ejaculatory processes absent; ejaculatory apodeme formed by single dorso-ventrally oriented plate; ventro-median margin of dorsal aedeagal sheath heavily sclerotised (appearing entirely closed); dorsal aedeagal sheath short, sperm sac entirely free; sperm sac appearing ± heavily sclerotised.

###### Description Female:

Unknown.

###### Material examined:

**Thailand:** Loei: 1 ♂ Phu Kradueng National Park, Forest protection unit Loei .5 (Phakbung), 16°50'32"N, 101°41'40"E, T1499, 13–19.ii.2007, malaise trap, Wuthicahi kwanjam (AAM-001138 1 ♂ holotype, LACM).

###### Type locality and distribution:

Phu Kradueng National Park (16°50'32"N, 101°41'40"E), Thailand (Fig. 46). Biodiversity hotspot/high-biodiversity wilderness area: Indo-Burma/-.

### Syllegomydinae

The Syllegomydinae is the most speciose subfamily taxon of Mydidae with currently 204 valid species in 25 genera. The species are distributed throughout Africa (northern Africa in the Palaearctic Region as well as sub-Saharan Africa in the Afrotropical Region), Israel, Madagascar, and Spain (see placement of representatives of Cephalocera Latreille, 1829 and Syllegomydas Becker, 1906 from India in the Discussion). It is by far the dominant taxon in southern Africa - the most speciose region for Mydidae world-wide (Hesse 1969, 1972, Bowden 1980).

#### Mydaselpidini

##### Mydaselpis

Bezzi, 1924

###### General.

The genus Mydaselpis comprises five valid species distributed in southern Africa (South Africa and Zimbabwe; Bezzi 1924, Hesse 1969, Bowden 1980) and representatives have not been reported from eastern Africa. The latest key to species was published by Hesse (1969: 22).

##### Mydaselpis ngurumani sp. n.

urn:lsid:zoobank.org:act:0B0B0FDD-643C-4921-979C-CB2B745EDA54

**Figures 10–20.**
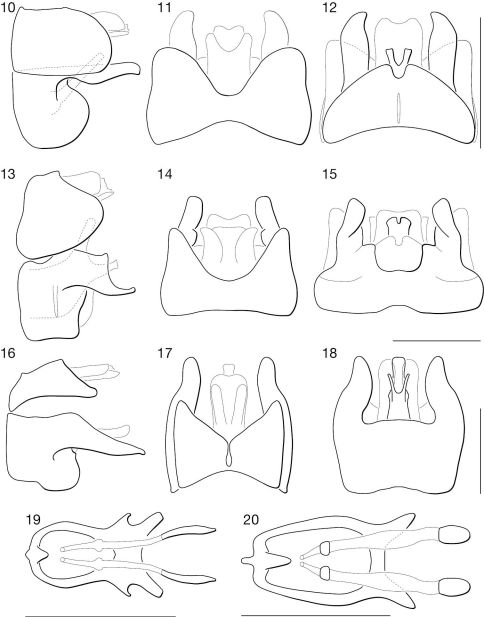
♂ terminalia and ♀ genitalia of Mydaselpis, Syllegomydas, and Vespiodes species. **10–18.** ♂ terminalia. **10–12.** Mydaselpis ngurumani sp. n. **10** lateral **11** dorsal **12** ventral. **13–15.** Vespiodes phaios sp. n. **13** lateral **14** dorsal **15** ventral. **16–18.** Syllegomydas heothinos sp. n. **16** lateral **17** dorsal **18** ventral. **19–20.** ♀ genitalia (posterior = left). **19** Mydaselpis ngurumani sp. n. dorsal **20** Syllegomydas heothinos sp. n. dorsal. Scale lines = 1 mm.

Figs 10–12, 19 33–34 43 47 

###### Etymology:

The specific epithet refers to the type locality of the Nguruman Escarpment in the Rift Valley Province, Kenya.

###### Diagnosis:

The species is distinguished from congeners by the parallel abdominal tergites 2–4 that do not form a vespiform waist, the reduced alula, the yellow and brown abdominal tergites in the male, the partly hyaline wings, features of the male terminalia, the presence of acanthophorite plates with spurs in the female ovipositor, and its apparent distribution in Kenya and Tanzania.

###### Description Male:

Head: brown, in general silver pruinose; width distinctly greater than thorax, interocular distance on vertex same as at ventral eye margin, vertex between compound eyes slightly depressed, parafacial area less than ½ the width of central facial gibbosity; facial gibbosity distinct, well-developed and discernible in lateral view; mystax white, covering only lateral facial gibbosity (asetose medially); frons medially apruinose, laterally grey pruinose, vertex apruinose, postgenae lightly silver pruinose; setation: vertex white, frons white, ocp setae white, pocl setae white; ocellar triangle apruinose; proboscis brown, long, reaching fronto-clypeal suture; labella large, much wider than prementum, as long as prementum, unsclerotised laterally; maxillary palpi cylindrical, brown, minute.

Antenna: brown, scape and pedicel white setose dorsally and ventrally; postpedicel cylindrical in proximal ½, symmetrically bulbous in distal ½, ≥ 8.0 times as long as combined length of scape and pedicel; apical ‚seta-like‘ sensory element situated apically in cavity on postpedicel.

Thorax: brown, predominantly apruinose; scutum medially dark brown, laterally brown, surface microrugose, predominantly apruinose, only lateral margins silver pruinose, scutal setation comprised of distinct rows of long dorsocentral setae and lateral scutal setae; dc setae pre- and postsuturally white, acr setae absent, lateral scutal setae white, npl, spal, and pal setae absent; postpronotal lobe light brown, silver pruinose; proepisternum, lateral postpronotum, and postpronotal lobes long white setose; scutellum silver pruinose proximally, apruinose distally, asetose, apical scutellar setae absent; mesopostnotum, anatergite, and katatergite silver pruinose, mesopostnotum asetose, anatergite asetose, katatergite long white setose; katatergite ± flat; anterior anepisternum asetose, supero-posterior anepisternum asetose; posterior anepimeron asetose, katepimeron asetose; metepimeron ± flat, yellow, silver pruinose, white setose; metepisternum silver pruinose, asetose.

Leg: light brown or yellow, setation predominantly brown; pro coxa apruinose, asetose, mes coxa apruinose, asetose, met coxa laterally silver pruinose; met trochanter setose medially; femora light brown or yellow, met femora evenly clubbed in distal ¾, in distal ½ macrosetose, 1 antero-ventral and 1 postero-ventral row of macrosetae; pro and mes tibiae laterally arched, met tibia straight, met tibia cylindrical, ventral keel absent; pro and mes tarsomere 1 about as long as individual tarsomeres 2, 3, or 4, met tarsomere 1 as long as combined length of tarsomeres 2–3; pulvilli well-developed, as long as well-developed claws, and as wide as base of claws; empodium absent.

Wing: length = 9.6–12.0 mm; slightly brown stained, darker brown around veins, veins brown, microtrichia absent; cells r_1_, r_4_, r_5_, m_3_, + cu*p* closed; C terminates at junction with R_1_; R_4_ terminates in R_1_; R_5_ terminates in R_1_; stump vein (R_3_) at base of R_4_ present, short not reaching R_2_; R_4_ and R_5_ widest apart medially; r-m distinct, R_4+5_ and M_1_ apart, connected by crossvein or indistinct, R_4+5_ and M_1_ fused; M_1_ straight at r-m (not curving anteriorly), M_1_ (or M_1_+M_2_) terminates in R_1_; CuA_1_ and CuA_2_ split proximally to m-cu (cell m_3_ narrow proximally); m_3_+CuA_1_ do not terminate together in C; A_1_ undulating, cell A_1_ wide, A_1_ and wing margin further apart proximally than distally, alula entirely reduced (nearly straight wing margin); halter brown.

Abdomen: brown and yellow; setation comprised of scattered brown setae, surface entirely smooth; T1 brown, T2–7 brown with yellow anterior and posterior margins; T1 brown setose, T2 anterior ½ asetose and posterior ½ brown setose, T3 anterior ¼ asetose and posterior ¾ brown setose; T predominantly apruinose; S1 brown, S2–7 brown with yellow anterior and posterior margins; S1 asetose, S2–3 anterior ½ asetose and posterior ½ brown setose; S predominantly apruinose; T2–4 parallel-sided and not constricted waist-like; bullae on T2 brown, transversely elongate, surface entirely smooth, T2 surface anterior to bullae smooth.

Male terminalia: T1–7 well-developed, entirely sclerotised, T8 medially weakly sclerotised, divided into 2 lateral heavily sclerotised sclerites; T7–8 anteriorly with 2 lateral apodemes; S6 regular, without any special setation postero-medially, S8 well-developed and simple, not fused to T8 dorso-laterally, entire (undivided) ventro-medially; epandrium formed by single sclerite (fused medially ± entirely), blunt, evenly rounded; subepandrial sclerite without lateral or median protuberances; hypandrium strongly concave, cup-shaped, entirely sclerotised ventrally (forming a single sclerite), entirely fused with gonocoxite, forming a gonocoxite-hypandrial complex; gonocoxites dorso-ventrally flattened (same height throughout, expanded laterally and medially), without median or lateral protuberance, gonocoxal apodeme absent; 2 functional aedeagal prongs, short and wide, medio-distally free, parallel or diverging laterally, distally straight or only diverging slightly laterally; aedeagal epimere absent; lateral ejaculatory processes absent; ejaculatory apodeme formed by single dorso-ventrally oriented plate; ventro-median margin of dorsal aedeagal sheath heavily sclerotised (appearing entirely closed); dorsal aedeagal sheath long, sperm sac entirely covered; sperm sac appearing ± heavily sclerotised.

###### Description Female:

Head: in general densely white pruinose; vertex between compound eyes ± horizontally straight, medially only slightly below dorsal eye margin; postgenae apruinose; pocl setae yellow.

Antenna: scape and pedicel brown setose dorsally and ventrally; postpedicel ≥ 9.0 times as long as combined length of scape and pedicel.

Thorax: scutum uniformly brown, surface entirely smooth, scutal setation comprised of scattered short white setae; proepisternum, lateral postpronotum, and postpronotal lobes short white setose; scutellum apruinose, asetose; metepimeron same colour as T1.

Leg: all coxae apruinose, brown setose; femora light brown; all tibiae laterally arched, pro and mes tarsomere 1 as long as combined length of tarsomeres 2–3.

Wing: length = 12.5–12.7 mm; r-m distinct, R_4+5_ and M_1_ apart, connected by crossvein.

Abdomen: brown; T1 entirely brown, T2 brown with anterior and posterior margins yellow, T3–4 brown with posterior margin yellow; T1–3 sparsely brown setose; S1 brown, S2 brown with anterior and posterior margins yellow, S3–4 brown with posterior margin yellow; S1 asetose, S2–3 sparsely brown setose; T2 surface anterior to bullae smooth.

Female genitalia: densely arranged anteriorly directed setae absent, only very few on T8 and S8; T8 with broad anterior rectangular apodeme; T9 formed by wide, rectangular sclerite with median protuberance; T9+10 entirely fused, T10 divided into 2 heavily sclerotised acanthophorite plates, 10 acanthophorite spurs per plate; 2 spermathecae, all equally large, not differentiated from spermathecal ducts, weakly sclerotised; individual spermathecal ducts short; S9 (furca) formed by 1 sclerite, ring-like (joined anteriorly and posteriorly), anterior furcal apodeme present, 2 lateral projections forming divided apodeme, lateral furcal apodeme present, median furcal bridge absent.

###### Material examined:

**Kenya:** Coast Province: 1 ♂ Watamu Mida Creek, 03°22'00"S, 039°57'00"E, 24.i.1987, A. Weaving (AAM-000142 paratype, AMGS); Rift Valley Province: 2 ♀ 2 ♂ Nguruma, 01°50'00"S, 036°56'00"E, -.vi.1990, I. Abu-Zinid (AAM-000149–AAM-000152 paratypes, NMSA); 3 ♂ Nguruman, 01°54'00"S, 036°02'00"E, 20.vi.1996, R. Copeland (AAM-000146 1 ♂ holotype, AAM-000143 + AAM-000145 paratypes, NMKE); 1 ♂ Nguruman, 2.vii.1996, R. Copeland (AAM-000144 paratype, NMKE); **Tanzania:** Kilimanjaro Region: 2 ♂ Same, 18 km S, 04°13'00"S, 037°46'00"E, 15.vii.2001, O. Haji W. Pulawski (AAM-000147–AAM-000148 paratypes, CAS).

###### Type locality and distribution:

Nguruman (01°54’S, 036°02’E), Kenya. Kenya, Tanzania (Fig. 47). Biodiversity hotspot/high-biodiversity wilderness area: Coastal Forest of Eastern Africa and Eastern Afromontane/-.

##### Vespiodes

Hesse, 1969

###### General.

To this day, nine species of Vespiodes are known from the Afrotropical Region occurring from Ghana in the north-west to north-eastern South Africa in the south (Sack 1935, Bequaert 1940, 1951, Hesse 1969, Bowden 1980), but representatives have not been reported from eastern Africa with the exception of central Malawi from where Vespiodes nyasae Hesse, 1969 was described. The latest key to species was published by Hesse (1969: 33).

##### Vespiodes phaios sp. n.

urn:lsid:zoobank.org:act:F1778548-C8BC-4EE4-A1	BF-0CB5923EFD96

Figs 13–15 35 48 

###### Etymology:

*phaios* Greek adjective = brown. Referring the overall brown coloration of this attractive species.

###### Diagnosis:

The species is distinguished from congeners by the predominantly apruinose and asetose scutum, the asetose abdominal tergites, features of the male terminalia, and its apparent distribution in south-eastern coastal Kenya.

###### Description Male:

Head: brown, in general silver pruinose; width distinctly greater than thorax, interocular distance on vertex larger than at ventral eye margin, vertex between compound eyes slightly depressed, parafacial area less than ½ the width of central facial gibbosity; facial gibbosity distinct, well-developed and discernible in lateral view; mystax white, covering only lateral facial gibbosity (asetose medially); frons predominantly apruinose, vertex apruinose, postgenae lightly silver pruinose; setation: vertex white, frons white, ocp setae brown or white, pocl setae white; ocellar triangle apruinose; proboscis brown, long, projecting beyond fronto-clypeal suture; labella large, much wider than prementum, about ½ length of prementum, unsclerotised laterally; maxillary palpi laterally compressed (leaflike), brown or light brown, shorter than length of pedicel.

Antenna: brown or orange, scape and pedicel white setose dorsally and ventrally; postpedicel cylindrical in proximal ½, symmetrically bulbous in distal ½, ≥ 11.0 times as long as combined length of scape and pedicel; apical ‚seta-like‘ sensory element situated apically in cavity on postpedicel.

Thorax: brown, predominantly apruinose; scutum medially dark brown, laterally brown, surface macrorugose, predominantly apruinose, small antero-lateral spots and extreme lateral margins (dorsal to supero-posterior anepisternum) silver pruinose, scutal setation comprised of distinct rows of long dorsocentral setae and lateral scutal setae; dc setae pre- and postsuturally white, acr setae absent, lateral scutal setae white, npl, spal, and pal setae absent; postpronotal lobe light brown, partly silver pruinose; proepisternum, lateral postpronotum, and postpronotal lobes long brown setose or long white setose; scutellum apruinose, asetose, apical scutellar setae absent; mesopostnotum, anatergite, and katatergite apruinose, mesopostnotum asetose, anatergite asetose, katatergite long white setose; katatergite ± flat; anterior anepisternum asetose, supero-posterior anepisternum asetose; posterior anepimeron asetose, katepimeron asetose; metepimeron evenly elevated, same colour as T1, grey pruinose, asetose; metepisternum grey pruinose, asetose.

Leg: brown, setation predominantly brown; pro coxa apruinose, short white setose, mes coxa apruinose, short white setose, met coxa grey pruinose, white setose; met trochanter setose medially; femora brown, met femur proximally yellow, met femora distinctly clubbed in distal ⅔, in distal ½ macrosetose, 1 antero-ventral and 1 postero-ventral row of macrosetae; all tibiae laterally arched, met tibia cylindrical, ventral keel absent; pro and mes tarsomere 1 as long as combined length of tarsomeres 2–3, met tarsomere 1 slightly longer than tarsomere 2, tarsomeres 1 and 2 longer than tarsomeres 3 and 4 combined; pulvilli well-developed, as long as well-developed claws, and as wide as base of claws; empodium absent.

Wing: length = 15.9 mm; slightly brown stained, darker brown stained anterior to and distal to M veins, veins brown, microtrichia absent; cells r_1_, r_4_, r_5_, m_3_, + cu*p* closed; C terminates at junction with R_1_; R_4_ terminates in R_1_; R_5_ terminates in R_1_; stump vein (R_3_) at base of R_4_ present, long but not reaching R_2_; R_4_ and R_5_ widest apart medially; r-m distinct, R_4+5_ and M_1_ apart, connected by crossvein; M_1_ curves anteriorly at r-m, M_1_ (or M_1_+M_2_) terminates in R_1_; CuA_1_ and CuA_2_ split proximally to m-cu (cell m_3_ narrow proximally); M_3_+CuA_1_ do not terminate together in C; A_1_ undulating, cell a_1_ wide, A_1_ and wing margin further apart proximally than distally, alula well-developed; halter brown.

Abdomen: brown and yellow; setation comprised of scattered white and brown setae, surface entirely smooth; T1 brown, T2–4 brown with yellow anterior margins; T1–3 sparsely white setose; T predominantly apruinose; S1 brown, S2–4 brown with yellow anterior margins; S1 asetose, S2–3 sparsely white setose; S predominantly apruinose; T2–4 distinctly narrowed, forming a waist, abdomen vespiform; bullae on T2 brown, transversely elongate, surface entirely smooth, T2 surface anterior to bullae smooth.

Male terminalia: T1–7 well-developed, entirely sclerotised, T8 postero-medially weakly sclerotised, with anterior transverse sclerotised bridge connecting lateral sclerites; T7–8 anteriorly with 2 lateral apodemes; S6 regular, without any special setation postero-medially, S8 well-developed and simple, not fused to T8 dorso-laterally, entire (undivided) ventro-medially; epandrium formed by single sclerite (fused medially ± entirely), blunt, evenly rounded; subepandrial sclerite without lateral or median protuberances; hypandrium strongly concave, cup-shaped, entirely sclerotised ventrally (forming a single sclerite), partially fused with gonocoxite anteriorly; gonocoxites dorso-ventrally flattened in distal ½, higher in proximal ½, without median or lateral protuberance, gonocoxal apodeme present, short (at most slightly extending hypopygium anteriorly); 2 functional aedeagal prongs, short and wide, medio-distally connected; aedeagal epimere present, distally simple, evenly rounded; lateral ejaculatory processes absent; ejaculatory apodeme formed by single dorso-ventrally oriented plate; ventro-median margin of dorsal aedeagal sheath heavily sclerotised (appearing entirely closed); dorsal aedeagal sheath long, sperm sac entirely covered; sperm sac appearing ± heavily sclerotised.

###### Description Female:

Unknown.

###### Material examined:

**Kenya:** Coast Province: 1 ♂ Muhaka Forest, 04°19'29"S; 039°31'27"E, 3.iii.2000, R. Copeland (AAM-000153 1 ♂ holotype, NMKE).

###### Type locality and distribution:

Muhaka Forest (04°19'29"S; 039°31'27"E), Kenya (Fig. 48). Biodiversity hotspot/high-biodiversity wilderness area: Coastal Forest of Eastern Africa/-.

#### Syllegomydini

##### Syllegomydas

Becker, 1906

###### General.

The genus Syllegomydas comprises 24 valid species distributed primarily in northern Africa, with a few species in sub-Saharan Africa (Chad, Mali, Malawi, Mozambique, Niger, Zimbabwe), as well as Afghanistan, Israel, and Spain (e.g., Arias 1914a, b, Bezzi 1924, Séguy 1941, Bequaert 1951, 1961, Hesse 1969, Bowden 1980, Carles-Tolrá 2006, see placement of Syllegomydas bucciferus Séguy, 1928 from India in the Discussion). The subgenus Syllegomydas (Notobates) Hesse, 1969 has been described for 2 species from southern Africa (Mozambique and Zimbabwe).

##### Syllegomydas (Syllegomydas) astrictus sp. n.

urn:lsid:zoobank.org:act:395C17A4-D779-458C-8944-BB0094D83DCA

Figs 21–23 36–37 47 

###### Etymology:

*astrictus* Latin adjective = drawn together. Referring to the narrow postgenae so that the compound eyes nearly touch each other ventrally.

###### Diagnosis:

The species is distinguished from congeners by the very narrow postgenae in the male so that the compound eyes nearly touch each other ventrally, the long white acrostichal setae in the male, the overall brown coloration in the female, the presence of lateral furcal apodemes in females, and its apparent distribution in Kenya.

###### Description Male:

Head: black, facial gibbosity light brown, in general densely white pruinose; width distinctly greater than thorax, interocular distance on vertex distinctly larger than at ventral eye margin, postgenae very narrow and eyes nearly touching ventrally, vertex between compound eyes slightly depressed, parafacial area about as wide as ½ the width of central facial gibbosity; facial gibbosity distinct, well-developed and discernible in lateral view; mystax white, covering entire facial gibbosity; frons medially apruinose, laterally grey pruinose, vertex medially apruinose, laterally grey pruinose, postgenae grey pruinose; setation: vertex white, frons white, ocp setae white, pocl setae white; ocellar triangle apruinose; proboscis light brown, short, about ½ length of oral cavity; labella small, as wide as prementum, only forming distal tip of proboscis, unsclerotised laterally; maxillary palpi cylindrical, light brown, minute.

Antenna: brown or orange, scape and pedicel white setose dorsally and ventrally; postpedicel cylindrical in proximal ½, symmetrically bulbous in distal ½, ≥ 4.0 times as long as combined length of scape and pedicel; apical ‚seta-like‘ sensory element situated apically in cavity on postpedicel.

Thorax: brown, predominantly grey pruinose; scutum medially bluish-black, laterally brown, surface entirely smooth, predominantly grey pruinose, broad sublateral stripes (interrupted postsuturally) and narrow paramedial stripes (merging postsuturally) apruinose, scutal setation comprised of distinct rows of long dorsocentral setae and lateral scutal setae; dc setae pre- and postsuturally white, acr setae present, lateral scutal setae white, npl, spal, and pal setae absent; postpronotal lobe light brown, grey pruinose; proepisternum, lateral postpronotum, and postpronotal lobes long white setose; scutellum grey pruinose proximally, apruinose distally, asetose, apical scutellar setae absent; mesopostnotum, anatergite, and katatergite grey pruinose, mesopostnotum laterally (close to anatergite) long white setose, anatergite long white setose, katatergite long white setose; katatergite ± flat; anterior anepisternum asetose, supero-posterior anepisternum long white setose; posterior anepimeron long white setose, katepimeron white setose; metepimeron ± flat, same colour as T1, grey pruinose, long white setose; metepisternum grey pruinose, asetose.

Leg: light brown, setation predominantly white; all coxae grey pruinose, white setose; met trochanter setose medially; femora light brown, met femora evenly clubbed in distal ¾, in distal ½ macrosetose, 1 antero-ventral and 1 postero-ventral row of macrosetae; all tibiae laterally arched, met tibia cylindrical, ventral keel absent; pro and mes tarsomere 1 about as long as individual tarsomeres 2, 3, or 4, met tarsomere 1 as long as combined length of tarsomeres 2–3; pulvilli well-developed, as long as well-developed claws, and as wide as base of claws; empodium absent.

Wing: length = 7.3–8.3 mm; hyaline throughout, veins light brown, microtrichia absent; cells r_1_, r_4_, r_5_, m_3_, + cup closed; C terminates at junction with R_1_; R_4_ terminates in R_1_; R_5_ terminates in R_1_; stump vein (R_3_) at base of R_4_ present, short not reaching R_2_; R_4_ and R_5_ widest apart medially; r-m distinct, R_4+5_ and M_1_ apart, connected by crossvein; M_1_ straight at r-m (not curving anteriorly), M_1_ (or M_1_+M_2_) terminates in R_1_; CuA_1_ and CuA_2_ split proximally to m-cu (cell m_3_ narrow proximally); M_3_+CuA_1_ do not terminate together in C; A_1_ undulating, cell A_1_ wide, A_1_ and wing margin further apart proximally than distally, alula well-developed; halter light yellow.

Abdomen: brown; setation comprised of scattered white setae, surface entirely smooth; T1–7 brown, yellow posterior margins; T1–2 and anterior ⅓ of T3 long white setose, remaining T3 brown setose; T brown pruinose proximally, grey pruinose distally; S1–7 light brown; S1 asetose, S2 long white setose, S3 short brown setose; S entirely grey pruinose; T2–4 tapering slightly posteriorly; bullae on T2 brown, transversely elongate, surface entirely smooth, T2 surface anterior to bullae smooth.

Male terminalia: T1–8 well-developed; T7–8 anteriorly with 2 lateral apodemes; S6 regular, without any special setation postero-medially, S8 well-developed and simple, not fused to T8 dorso-laterally, entire (undivided) ventro-medially; epandrium formed by single sclerite (fused medially ± entirely), blunt, evenly rounded; subepandrial sclerite without lateral or median protuberances; hypandrium strongly concave, cup-shaped, entirely sclerotised ventrally (forming a single sclerite), entirely fused with gonocoxite, forming a gonocoxite-hypandrial complex; gonocoxites dorso-ventrally flattened (same height throughout, expanded laterally and medially), without median or lateral protuberance, gonocoxal apodeme absent; 2 functional aedeagal prongs, short and wide, medio-distally free, parallel or diverging laterally, distally straight or only diverging slightly laterally; aedeagal epimere present, distally simple, evenly rounded; lateral ejaculatory processes absent; ejaculatory apodeme formed by single dorso-ventrally oriented plate; ventro-median margin of dorsal aedeagal sheath heavily sclerotised (appearing entirely closed); dorsal aedeagal sheath long, sperm sac entirely covered; sperm sac appearing ± heavily sclerotised.

###### Description Female:

Head: brown, in general grey pruinose; interocular distance on vertex larger than at ventral eye margin, vertex between compound eyes ± horizontally straight, medially only slightly below dorsal eye margin; parafacial area more than ½ the width of central facial gibbosity; mystax white, covering only lateral facial gibbosity (asetose medially); postgenae apruinose; pocl setae yellow.

Antenna: scape and pedicel white and yellow setose dorsally and ventrally.

Thorax: light brown, scutum medially brown, laterally light brown, predominantly grey pruinose, broad sublateral stripes (interrupted postsuturally) and narrow paramedial stripes (not reaching posterior margin) apruinose, scutal setation comprised of scattered short white setae; proepisternum, lateral postpronotum, and postpronotal lobes short white setose; supero-posterior anepisternum short white setose; anatergite short white setose; katatergite short white setose.

Leg: met femora ± cylindrical only slightly wider than pro and mes femora; pro and mes tibiae laterally arched, met tibia straight; met tarsomere 1 longer than combined length of tarsomeres 2–4; pulvilli reduced, half length of well-developed claws.

Wing: length = 9.3–10.6 mm; slightly brown stained, darker brown around veins; halter light brown.

Abdomen: setation comprised of sparsely scattered short yellow setae, T1–3 sparsely yellow setose; T predominantly apruinose; S1 asetose, S2–3 sparsely yellow setose; S predominantly apruinose; T2–4 parallel-sided and not constricted waist-like; bullae on T2 brown, oval.

Female genitalia: densely arranged anteriorly directed setae present on T5–8 and S5–8; T8 with broad anterior rectangular apodeme; T9 formed by wide, rectangular sclerite with median protuberance; T9+10 entirely fused, T10 divided into 2 heavily sclerotised acanthophorite plates, 10 acanthophorite spurs per plate; 2 spermathecae, all equally large, formed by ± expanded weakly sclerotised ducts; individual spermathecal ducts short; S9 (furca) formed by 1 sclerite, ring-like (joined anteriorly and posteriorly), anterior furcal apodeme present, 2 lateral projections forming divided apodeme, lateral furcal apodeme present, median furcal bridge absent.

**Figures 21–29.**
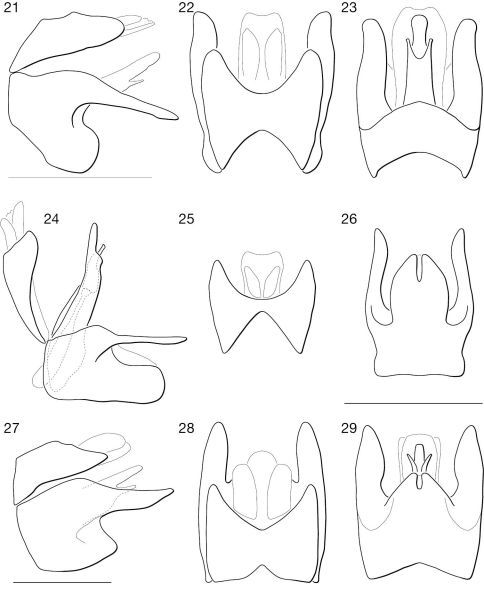
♂ terminalia of Syllegomydas species. **21–23.** Syllegomydas astrictus sp. n. **21** lateral **22** dorsal **23** ventral. **24–26.** Syllegomydas elachys sp. n. **24** lateral **25** dorsal **26** ventral. **27–29.** Syllegomydas dispar **27** lateral **28** dorsal **29** ventral. Scale lines = 1 mm.

###### Material examined:

**Kenya:** Eastern Province: 2 ♂ Isiolo, 5 km NNE, 00°24'18"N; 037°35'42"E, 8–10.vi.2000, M. Bourbon V. Lee W. Pulawski (AAM-000154–AAM-000155 paratypes, CAS); 1 ♂ Athi River, 02°38'31"N; 038°21'59"E, Malaise Trap, 5–10.vii.1999, R. Copeland (AAM-000170 paratype, NMKE); 1 ♀ Nguruman near Sampu River, 01°54'04"S; 036°02'53"E, 753 m, 17.vi.1997, R. Copeland (AAM-001125 paratype, NMKE); 9 ♂ Nguruman near Sampu River, Malaise Trap near Nguruman Escarpment, 4–18.viii.2007, R. Copeland (AAM-000164 1 ♂ holotype, AAM-000161–AAM-000163, AAM-000165–AAM-000169 paratypes, NMKE); Rift Valley Province: 1 ♀ Magadi Road, 46 air km SW Nairobi, 01°34'00"S; 036°27'24"E, 29.vi.1999, W. Pulawski J. Schweikert (AAM-000156 paratype, CAS); 4 ♂ Chyulu Hills, 02°36'00"S; 037°51'00"E, Malaise Trap, 1–8.vii.2006, R. Copeland (AAM-000157–AAM-000160 paratypes, NMKE).

**Figures 30–41.**
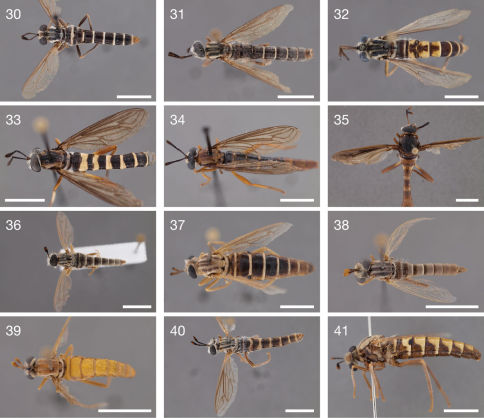
Photographs of newly described species. **30** Leptomydas notos sp. n. (♂ holotype, AAM-000776, AMNH) **31** Leptomydas rapti sp. n. (♂ holotype, AAM-000115, ZSMC) **32** Leptomydas tigris sp. n. (♂ holotype, AAM-001138, LACM) **33** Mydaselpis ngurumani sp. n. (♂ holotype, AAM-000146, NMKE) **34** Mydaselpis ngurumani sp. n. (♀ paratype, AAM-000152, NMSA) **35** Vespiodes phaios sp. n. (♂ holotype, AAM-000153, NMKE) **36** Syllegomydas (Notobates) astrictus sp. n. (♂ holotype, AAM-000164, NMKE) **37** Syllegomydas (Notobates) astrictus sp. n. (♀ paratype, AAM-000156, CAS) **38** Syllegomydas (Syllegomydas) elachys sp. n. (♂ paratype, AAM-001124, ZSMC) **39** Syllegomydas (Syllegomydas) elachys sp. n. (♀ paratype, AAM-001118, ZSMC) **40** Syllegomydas (Notobates) heothinos sp. n. (♂ holotype, AAM-001126, NMKE) **41** Syllegomydas (Notobates) heothinos sp. n. (♀ paratype, AAM-000138, BMNH). Scale lines = 5 mm.

**Figures 42–45.**
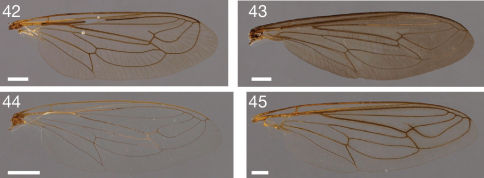
Photographs of wings. **42** Leptomydas notos sp. n. (♂ paratype, AAM-000770, AMNH) **43** Mydaselpis ngurumani sp. n. (♂ paratype, AAM-000145, NMKE) **44** Syllegomydas (Syllegomydas) elachys sp. n. (♂ paratype, AAM-001114, ZSMC) **45** Syllegomydas (Notobates) heothinos sp. n. (♂ paratype, AAM-001104, BMNH). Scale lines = 1 mm.

###### Type locality and distribution:

Nguruman near Sampu River (01°54'04"S; 036°02'53"E), Kenya (Fig. 47). Biodiversity hotspot/high-biodiversity wilderness area: -/-.

##### Syllegomydas (Syllegomydas) elachys sp. n.

urn:lsid:zoobank.org:act:9A0DBEE7-3AF1-404D-95DB-E0440AA07719

Figs 24–26 38–39 44 48 

###### Etymology:

*elachys* Greek adjective = little, small. Referring to the small size of this species.

###### Diagnosis:

The species is distinguished from congeners by the very small size, the entirely grey pruinose abdominal tergites, the overall light brown coloration in females, the relatively short antennae, the very short proboscis, the parallel longitudinal ridges on the bullae, the presence of lateral furcal apodemes in females, and its apparent distribution in northern Zimbabwe.

###### Description Male:

Head: brown, in general densely grey pruinose; width distinctly greater than thorax, interocular distance on vertex larger than at ventral eye margin, vertex between compound eyes ± horizontally straight, medially only slightly below dorsal eye margin, parafacial area more than ½ the width of central facial gibbosity; facial gibbosity distinct, well-developed and discernible in lateral view; mystax light brown, covering entire facial gibbosity or white, covering entire facial gibbosity; frons entirely grey pruinose, vertex entirely grey pruinose, postgenae apruinose or lightly silver pruinose; setation: vertex light brown or white, frons light brown or white, ocp setae white, pocl setae white; ocellar triangle apruinose; proboscis light brown, very short, vestigial, knob-like; labella small, as wide as prementum, only forming distal tip of proboscis, unsclerotised laterally; maxillary palpi cylindrical, light brown, slightly longer than proboscis.

Antenna: brown or orange, scape and pedicel brown setose dorsally and ventrally; postpedicel cylindrical in proximal ½, symmetrically bulbous in distal ½, ≥ 4.0 times as long as combined length of scape and pedicel; apical ‚seta-like‘ sensory element situated apically in cavity on postpedicel.

Thorax: brown, predominantly grey pruinose; scutum medially bluish-black, laterally brown, surface entirely smooth, predominantly grey pruinose, asetose median and sublateral stripes sparsely grey pruinose, setose areas densely grey pruinose, scutal setation comprised of distinct rows of long dorsocentral setae and lateral scutal setae; dc setae pre- and postsuturally white, acr setae absent, lateral scutal setae white, npl, spal, and pal setae absent; postpronotal lobe light brown, grey pruinose; proepisternum, lateral postpronotum, and postpronotal lobes long white setose; scutellum entirely grey pruinose, asetose, apical scutellar setae absent; mesopostnotum, anatergite, and katatergite grey pruinose, mesopostnotum asetose, anatergite long white setose, katatergite long white setose; katatergite ± flat; anterior anepisternum asetose, supero-posterior anepisternum long white setose; posterior anepimeron long white setose, katepimeron white setose; metepimeron ± flat, same colour as T1, grey pruinose, long white setose; metepisternum grey pruinose, asetose.

Leg: light brown or yellow, setation predominantly white; all coxae grey pruinose, white setose; met trochanter setose medially; femora brown or light brown, met femora ± cylindrical only slightly wider than pro and mes femora, in distal ½ macrosetose, 1 antero-ventral and 1 postero-ventral row of macrosetae; all tibiae laterally arched, met tibia cylindrical, ventral keel absent; pro and mes tarsomere 1 as long as combined length of tarsomeres 2–3, met tarsomere 1 as long as combined length of tarsomeres 2–4; pulvilli well-developed, as long as well-developed claws, and as wide as base of claws; empodium absent.

Wing: length = 5.6–7.5 mm; hyaline throughout, veins light brown, microtrichia absent; cells r_1_, r_4_, r_5_, m_3_, + cu*p* closed; C terminates at junction with R_1_; R_4_ terminates in R_1_; R_5_ terminates in R_1_; stump vein (R_3_) at base of R_4_ present, short not reaching R_2_; R_4_ and R_5_ ± parallel medially; r-m distinct, R_4+5_ and M_1_ apart, connected by crossvein; M_1_ straight at r-m (not curving anteriorly), M_1_ (or M_1_+M_2_) terminates in R_1_; CuA_1_ and CuA_2_ split proximally to m-cu (cell m_3_ narrow proximally); m_3_+CuA_1_ do not terminate together in C; A_1_ undulating, cell A_1_ wide, A_1_ and wing margin further apart proximally than distally, alula well-developed; halter light brown.

Abdomen: brown; setation comprised of scattered white setae, surface entirely smooth; T1–7 brown, yellow posterior margins; T1 and anterior ¼ of T2 long white setose, remaining T2 and T3 brown and white setose; T entirely grey pruinose; S1–7 brown; S1 asetose, S2–3 sparsely yellow setose; S entirely grey pruinose; T2–4 parallel-sided and not constricted waist-like; bullae on T2 brown, transversely elongate, surface with parallel longitudinal ridges, T2 surface anterior to bullae smooth.

Male terminalia: T1–8 well-developed; T7–8 anteriorly with 2 lateral apodemes; S6 regular, without any special setation postero-medially, S8 well-developed and simple, not fused to T8 dorso-laterally, entire (undivided) ventro-medially; epandrium formed by single sclerite (fused medially ± entirely), rounded postero-laterally; subepandrial sclerite without lateral or median protuberances; hypandrium strongly concave, cup-shaped, entirely sclerotised ventrally (forming a single sclerite), entirely fused with gonocoxite, forming a gonocoxite-hypandrial complex; gonocoxites dorso-ventrally flattened in distal ½, higher in proximal ½, without median or lateral protuberance, gonocoxal apodeme absent; 2 functional aedeagal prongs, short and wide, medio-distally free, parallel or diverging laterally, distally straight or only diverging slightly laterally; aedeagal epimere present, distally simple, evenly rounded; lateral ejaculatory processes absent; ejaculatory apodeme formed by single dorso-ventrally oriented plate; ventro-median margin of dorsal aedeagal sheath heavily sclerotised (appearing entirely closed); dorsal aedeagal sheath long, sperm sac entirely covered; sperm sac appearing ± heavily sclerotised.

###### Description Female:

Head: brown, facial gibbosity light brown; parafacial area about as wide as ½ the width of central facial gibbosity.

Antenna: postpedicel ≥ 3.0 times as long as combined length of scape and pedicel.

Thorax: scutum uniformly brown; proepisternum, lateral postpronotum, and postpronotal lobes short white setose; anatergite short white setose; katatergite short white setose; supero-posterior anepisternum asetose; posterior anepimeron short white setose.

Leg: brown, setation brown and white; all coxae grey pruinose, brown setose; pro and mes tarsomere 1 longer than tarsomere 2, but less than combined length of tarsomeres 2–3.

Wing: length = 8.9–9.2 mm; slightly brown stained throughout, veins brown; halter brown.

Abdomen: setation comprised of sparsely scattered short brown setae, T1–7 brown; T1–3 sparsely brown setose; T predominantly apruinose; S1–3 asetose; S predominantly apruinose; bullae on T2 light brown, transversely elongate.

Female genitalia: densely arranged anteriorly directed setae absent, only few on T7–8 and S7–8; T8 with broad anterior rectangular apodeme; T9 formed by wide, rectangular sclerite with median protuberance; T9+10 entirely fused, T10 divided into 2 heavily sclerotised acanthophorite plates, 5–7 acanthophorite spurs per plate; 2 spermathecae, all equally large, formed by ± expanded weakly sclerotised ducts and spherical weakly sclerotised reservoirs; individual spermathecal ducts short; S9 (furca) formed by 1 sclerite, ring-like (joined anteriorly and posteriorly), anterior furcal apodeme present, 2 lateral projections forming divided apodeme, lateral furcal apodeme present, median furcal bridge absent.

###### Material examined:

**Zimbabwe:** Mashonaland East: 1 ♂ Kotwa, Broken Causeway, 17°03'00"S; 032°46'00"E, 7.ix.1986, M. Lillig S. Potel (AAM-001111 paratype, ZSMC); 1 ♂ Kotwa, Broken Causeway, 8.ix.1986, M. Lillig S. Potel (AAM-001112 paratype, ZSMC); 1 ♂ Kotwa, Broken Causeway, 31.viii.1986, M. Lillig S. Potel (AAM-001113 paratype, ZSMC); 1 ♂ Kotwa, Broken Causeway, 25.viii.1986, M. Lillig S. Potel (AAM-001114 paratype, ZSMC); 1 ♀ Kotwa, Chimana Causeway, 17°06'00"S; 032°38'00"E, 26.viii.1986, M. Lillig S. Potel (AAM-001115 paratype, ZSMC); Mashonaland West: 1 ♀ 3 ♂ Rukommetje Research Station, 16°06'00"S; 029°15'00"E, -.viii.1988, J. Weyrich (AAM-001117 1 ♂ holotype, AAM-001116, AAM-001118–AAM-001119 paratypes, ZSMC); 1 ♂ Rukommetje Research Station, -.viii.1988, J. Weyrich (AAM-001120 paratype, ZSMC); 4 ♂ SE Angwe Bridge, 16°05'00"S; 030°09'00"E, -.viii.1988, J. Weyrich (AAM-001121–AAM-001124 paratypes, ZSMC).

###### Type locality and distribution:

Rukommetje Research Station (16°06'00"S; 029°15'00"E), Zimbabwe (Fig. 48). Biodiversity hotspot/high-biodiversity wilderness area: -/Miombo-Mopane Woodlands and Savannas.

###### Remarks:

This species does not belong to the subgenus Notobates as defined by Hesse (1969: 274) and it might therefore present the southernmost distribution of the primarily Palaearctic subgenus Syllegomydas. The monophyly of Syllegomydas (Notobates) needs to be investigated and will be tested with a phylogenetic study of Mydidae currently in preparation by the author.

##### Syllegomydas (Syllegomydas) proximus

Séguy, 1928

###### Diagnosis:

The species is distinguished from congeners by the overall dark coloration in the male, the narrow yellow bands on the posterior margin of the abdominal tergites, the dark patterning of the female abdominal tergites, and its apparent distribution on the southern and western edges of the Sahara.

###### Re-description Male:

Head: brown, in general grey pruinose; width distinctly greater than thorax, interocular distance on vertex larger than at ventral eye margin, vertex between compound eyes slightly depressed, parafacial area less than ½ the width of central facial gibbosity; facial gibbosity distinct, well-developed and discernible in lateral view; mystax white, covering entire facial gibbosity; frons medially apruinose, laterally grey pruinose, vertex medially apruinose, laterally grey pruinose, postgenae apruinose; setation: vertex white, frons white, ocp setae white, pocl setae yellow; ocellar triangle apruinose; proboscis light brown, very short, vestigial, knob-like; labella small, as wide as prementum, only forming distal tip of proboscis, unsclerotised laterally; maxillary palpi cylindrical, light brown, slightly longer than proboscis.

Antenna: brown, scape and pedicel white setose dorsally, brown setose ventrally; postpedicel cylindrical in proximal ½, symmetrically bulbous in distal ½, ≥ 3.0 times as long as combined length of scape and pedicel; apical ‚seta-like‘ sensory element situated apically in cavity on postpedicel.

Thorax: light brown, predominantly grey pruinose; scutum uniformly brown, surface entirely smooth, predominantly grey pruinose, broad sublateral and median stripes (not reaching posterior margin) brown pruinose, scutal setation comprised of distinct rows of long dorsocentral setae and lateral scutal setae; dc setae pre- and postsuturally white, acr setae absent, lateral scutal setae white, npl, spal, and pal setae absent; postpronotal lobe yellow, white pruinose; proepisternum, lateral postpronotum, and postpronotal lobes long white setose; scutellum entirely grey pruinose, asetose, apical scutellar setae absent; mesopostnotum, anatergite, and katatergite grey pruinose, mesopostnotum laterally (close to anatergite) long white setose, anatergite long white setose, katatergite long white setose; katatergite ± flat; anterior anepisternum asetose, supero-posterior anepisternum long white setose; posterior anepimeron long white setose, katepimeron white setose; metepimeron ± flat, yellow, grey pruinose, long white setose; metepisternum grey pruinose, asetose.

Leg: brown, setation brown and white; all coxae grey pruinose, brown setose; met trochanter setose medially; femora brown, met femora ± cylindrical only slightly wider than pro and mes femora, in distal ½ macrosetose, 1 antero-ventral and 1 postero-ventral row of macrosetae; pro and mes tibiae laterally arched, met tibia straight, met tibia cylindrical, ventral keel absent; pro and mes tarsomere 1 longer than tarsomere 2, but less than combined length of tarsomeres 2–3, met tarsomere 1 as long as combined length of tarsomeres 2–4; pulvilli well-developed, as long as well-developed claws, and as wide as base of claws; empodium absent.

Wing: length = 9.0–9.7 mm; hyaline throughout, veins light brown or light yellow, microtrichia absent; cells r_1_, r_4_, r_5_, m_3_, + cu*p* closed; C terminates at junction with R_1_; R_4_ terminates in R_1_; R_5_ terminates in R_1_; stump vein (R_3_) at base of R_4_ absent or only very short; R_4_ and R_5_ widest apart medially; r-m distinct, R_4+5_ and M_1_ apart, connected by crossvein; M_1_ straight at r-m (not curving anteriorly), M_1_ (or M_1_+M_2_) terminates in R_1_; CuA_1_ and CuA_2_ split proximally to m-cu (cell m_3_ narrow proximally); m_3_+CuA_1_ do not terminate together in C; A_1_ undulating, cell A_1_ wide, A_1_ and wing margin further apart proximally than distally, alula well-developed; halter light brown.

Abdomen: brown; setation comprised of scattered white and brown setae, surface entirely smooth; T1–T7 brown with narrow yellow posterior margins; T1 and anterior ½ of T2 long white setose, remaining T2 and T3 brown setose; T lightly grey pruinose; S1–7 brown, yellow posterior margins; S1 asetose, S2–3 sparsely brown setose; S predominantly apruinose; T2–4 parallel-sided and not constricted waist-like; bullae on T2 light brown, small and circular, surface entirely smooth, T2 surface anterior to bullae smooth.

Male terminalia: T1–7 well-developed, entirely sclerotised, T8 postero-medially weakly sclerotised, with anterior transverse sclerotised bridge connecting lateral sclerites; T7–8 anteriorly with 2 lateral apodemes; S6 regular, without any special setation postero-medially, S8 well-developed and simple, not fused to T8 dorso-laterally, entire (undivided) ventro-medially; epandrium formed by single sclerite (fused medially ± entirely), rounded postero-laterally; subepandrial sclerite without lateral or median protuberances; hypandrium strongly concave, cup-shaped, entirely sclerotised ventrally (forming a single sclerite), entirely fused with gonocoxite, forming a gonocoxite-hypandrial complex; gonocoxites dorso-ventrally flattened in distal ½, higher in proximal ½, without median or lateral protuberance, gonocoxal apodeme absent; 2 functional aedeagal prongs, short and wide, medio-distally free, parallel or diverging laterally, distally straight or only diverging slightly laterally; aedeagal epimere present, distally simple, evenly rounded; lateral ejaculatory processes absent; ejaculatory apodeme formed by single dorso-ventrally oriented plate; ventro-median margin of dorsal aedeagal sheath heavily sclerotised (appearing entirely closed); dorsal aedeagal sheath long, sperm sac entirely covered; sperm sac appearing ± heavily sclerotised.

###### Re-description Female:

Head: in general densely grey pruinose; vertex between compound eyes ± horizontally straight, medially only slightly below dorsal eye margin; parafacial area more than ½ the width of central facial gibbosity; mystax white, covering entire facial gibbosity, sparse; pocl setae white.

Antenna: scape and pedicel white setose dorsally and ventrally; postpedicel ≥ 4.0 times as long as combined length of scape and pedicel.

Thorax: scutum uniformly light brown; mesopostnotum laterally (close to anatergite) short white setose; anatergite short white setose; katatergite short white setose; posterior anepimeron short white setose; supero-posterior anepisternum short white setose; metepimeron same colour as T1, white pruinose, short white setose.

Leg: light brown, setation predominantly white; all coxae grey pruinose, white setose; femora light brown or yellow; pulvilli reduced, half length of well-developed claws.

Wing: length = 11.2–11.5 mm; r-m distinct, R_4+5_ and M_1_ apart, connected by crossvein.

Abdomen: light brown; setation comprised of sparsely scattered short yellow setae, T1 light brown, T2–7 light brown with brown lateral longitudinal and sublateral circular markings; T1–3 sparsely yellow setose; T1–2 anteriorly lightly grey pruinose, T3–7 apruinose; S1 brown, S2–7 light brown with brown lateral longitudinal and median circular markings; S1–3 asetose.

Female genitalia: densely arranged anteriorly directed setae present on T7–8 and S7–8; T9 formed by wide, rectangular sclerite with median protuberance; T9+10 entirely fused, T10 divided into 2 heavily sclerotised acanthophorite plates, 6–8 acanthophorite spurs per plate; 2 spermathecae, all equally large, formed by ± expanded weakly sclerotised ducts and spherical weakly sclerotised reservoirs; individual spermathecal ducts short; S9 (furca) formed by 1 sclerite, ring-like (joined anteriorly and posteriorly), anterior furcal apodeme present, 2 lateral projections forming divided apodeme, lateral furcal apodeme present, median furcal bridge absent.

###### Material examined:

**Niger:** Niamey: 1 ♂ Niamey, 13°31'00"N; 002°07'00"E, -.xii.1938, L. Chepard (AAM-001219, MNHN); 3 ♂ Niamey, 16.xi.1977, millet field, J. Ritchie (AAM-000140, AAM-000663–AAM-000664, BMNH); Zinder: 3 ♀ 4 ♂ Dungass (= Dengas), 13°04'05"N; 009°20'23"E, -.xi.1910, R. Gaillard (1 ♂ holotype, AAM-001220–AAM-001225, MNHN); **Mauritania:** Trarza: 1 ♂ Nouakchott, 153 km NE, 18°59'45"N; 015°13'56"W, 20.x.1993, W. Pulawski (AAM-000116, CAS).

###### Distribution:

Niger, Mauritania. Biodiversity hotspot/high-biodiversity wilderness area: -/-.

###### Remarks:

The ♂ specimen (AAM-000116) of this small species from the western edges of the Sahara represents the first Mydidae species recorded from Mauritania. The species is primarily known from specimens collected in southern Niger (see material examined above that was first mentioned by Séguy 1928, 1941). It has also been recorded from Mali (Bowden 1980: 333) and North Africa (Sack 1934: 26). The specimen that Bowden based his assessment on has not been found in the BMNH so that I cannot verify the record from Mali. The northern African distribution is also questionable as this species has not been listed in the Palaearctic catalogue (Richter and Zaitzev 1988).

##### Syllegomydas (Notobates)

Hesse, 1969

Hesse (1969: 274) described the subgenus Notobates to accommodate two species, Syllegomydas arnoldi Bequaert, 1938 and Syllegomydas rhodesiensis Bequaert, 1938, from Mozambique and Zimbabwe and provided a key to the species. He did not place Syllegomydas dispar (Loew, 1852), also from Mozambique, in this subgenus, because he had not studied specimens of this species. Having studied the type specimens of Syllegomydas dispar at the ZMHB as well as additional specimens from several collections, it becomes clear that this species should also be placed in Syllegomydas (Notobates) based, for example, on the undulating vein A_1_ and the long antennae (longer than scutum). Both Hesse (1969: 295) and Bowden (1980: 333) listed Syllegomydas dispar as Syllegomydini *incertae sedis*. Therefore, this species is here re-described and illustrated to allow identification in the future.

##### Syllegomydas (Notobates) dispar

(Loew, 1852) comb. n.

Figs 27–29 47 

###### Diagnosis:

The species is distinguished from congeners by the brown and yellow abdominal tergites in males and females, the long antennae, the presence of long acrostichal setae, the yellow metepimeron, which is coloured differently than the adjacent abdominal tergite 1, the absence of lateral furcal apodemes in females, and its apparent distribution in south-eastern lowland and coastal Mozambique.

###### Re-description Male:

Head: brown, in general grey pruinose; width distinctly greater than thorax, interocular distance on vertex larger than at ventral eye margin, vertex between compound eyes ± horizontally straight, medially only slightly below dorsal eye margin, parafacial area less than ½ the width of central facial gibbosity; facial gibbosity distinct, well-developed and discernible in lateral view; mystax white, covering entire facial gibbosity; frons medially apruinose, laterally grey pruinose, vertex medially apruinose, laterally grey pruinose, postgenae apruinose; setation: vertex white, frons white, ocp setae white, pocl setae white; ocellar triangle apruinose; proboscis light brown, very short, vestigial, knob-like; labella small, as wide as prementum, as long as prementum, unsclerotised laterally; maxillary palpi cylindrical, light brown, slightly longer than proboscis.

Antenna: brown, scape and pedicel white setose dorsally and ventrally; postpedicel cylindrical in proximal ⅔, symmetrically bulbous in distal ⅓, ≥ 7.0 times as long as combined length of scape and pedicel; apical ‚seta-like‘ sensory element situated apically in cavity on postpedicel.

Thorax: dark brown to bluish-black, predominantly grey pruinose; scutum medially bluish-black, laterally brown, surface entirely smooth, predominantly grey pruinose, only broad sublateral stripes and narrow paramedial stripes (not reaching posterior margin) apruinose, scutal setation comprised of distinct rows of long dorsocentral setae and lateral scutal setae; dc setae pre- and postsuturally white, acr setae present, lateral scutal setae white, npl, spal, and pal setae absent; postpronotal lobe light brown, grey pruinose; proepisternum, lateral postpronotum, and postpronotal lobes long white setose; scutellum apruinose, asetose, apical scutellar setae absent; mesopostnotum, anatergite, and katatergite partly grey pruinose, anatergite and katatergite apruinose, mesopostnotum entirely long white setose, anatergite long white setose, katatergite long white setose; katatergite ± flat; anterior anepisternum asetose, supero-posterior anepisternum long white setose; posterior anepimeron long white setose, katepimeron white setose; metepimeron ± flat, yellow, apruinose, long white setose; metepisternum grey pruinose, asetose.

Leg: brown or light brown, setation predominantly white; all coxae apruinose, long white setose; met trochanter setose medially; femora brown, met femora ± cylindrical only slightly wider than pro and mes femora, in distal ½ macrosetose, 1 antero-ventral and 1 postero-ventral row of macrosetae; pro and mes tibiae laterally arched, met tibia straight, met tibia cylindrical, ventral keel absent; pro and mes tarsomere 1 about as long as individual tarsomeres 2, 3, or 4, met tarsomere 1 as long as combined length of tarsomeres 2–3 or less than combined length of tarsomeres 2–3; pulvilli well-developed, as long as well-developed claws, and as wide as base of claws; empodium absent.

Wing: length = 9.5–11.8 mm; slightly brown stained throughout, veins brown, microtrichia absent; cells r_1_, r_4_, r_5_, m_3_, + cu*p* closed; C terminates at junction with R_1_; R_4_ terminates in R_1_; R_5_ terminates in R_1_; stump vein (R_3_) at base of R_4_ present, short not reaching R2; R_4_ and R_5_ widest apart medially; r-m distinct, R_4_+5 and M_1_ apart, connected by crossvein; M_1_ straight at r-m (not curving anteriorly), M_1_ (or M_1_+M_2_) terminates in R_1_; CuA_1_ and CuA_2_ split proximally to m-cu (cell m_3_ narrow proximally); m_3_+CuA_1_ do not terminate together in C; A_1_ undulating, cell A_1_ wide, A_1_ and wing margin further apart proximally than distally, alula well-developed; halter light brown.

Abdomen: brown and yellow; setation comprised of scattered white and brown setae, surface entirely smooth; T1 brown, narrow yellow posterior margin, T2–7 brown, broad yellow posterior margins, expanding antero-laterally particularly on T2–3; T1 and anterior ½ of T2 long white setose, remaining T2 and T3 brown setose; T predominantly apruinose; S1–7 brown, yellow posterior margins; S1 asetose, S2 long white setose, S3 short white setose; S predominantly apruinose; T2–4 parallel-sided and not constricted waist-like; bullae on T2 brown, oval, surface entirely smooth, T2 surface anterior to bullae smooth.

Male terminalia: T1–7 well-developed, entirely sclerotised, T8 postero-medially weakly sclerotised, with anterior transverse sclerotised bridge connecting lateral sclerites; T7–8 anteriorly with 2 lateral apodemes; S6 regular, without any special setation postero-medially, S8 well-developed and simple, not fused to T8 dorso-laterally, entire (undivided) ventro-medially; epandrium formed by single sclerite (fused medially ± entirely), pointed postero-laterally; subepandrial sclerite without lateral or median protuberances; hypandrium strongly concave, cup-shaped, entirely sclerotised ventrally (forming a single sclerite), entirely fused with gonocoxite, forming a gonocoxite-hypandrial complex; gonocoxites dorso-ventrally flattened in distal ½, higher in proximal ½, without median or lateral protuberance, gonocoxal apodeme absent; 2 functional aedeagal prongs, short and wide, medio-distally free, parallel or diverging laterally, distally straight or only diverging slightly laterally; aedeagal epimere present, distally simple, evenly rounded; lateral ejaculatory processes absent; ejaculatory apodeme formed by single dorso-ventrally oriented plate; ventro-median margin of dorsal aedeagal sheath heavily sclerotised (appearing entirely closed); dorsal aedeagal sheath long, sperm sac entirely covered; sperm sac appearing ± heavily sclerotised.

###### Re-description Female:

Head: parafacial area about as wide as ½ the width of central facial gibbosity; maxillary palpi brown.

Thorax: brown, scutum medially brown, laterally light brown, predominantly grey pruinose, broad sublateral stripes (interrupted postsuturally) and narrow paramedial stripes (not reaching posterior margin) apruinose, scutal setation comprised of distinct rows of short dorsocentral setae and lateral scutal setae; proepisternum, lateral postpronotum, and postpronotal lobes short white setose; katatergite short white setose; supero-posterior anepisternum short white setose; metepimeron light brown.

Leg: all tibiae laterally arched.

Wing: length = 10.2 mm.

Abdomen: T1 brown, narrow yellow posterior margin, T2–7 brown, broad yellow posterior margins that are widest medially; T1 and anterior ¼ of T2 long white setose, remaining T2 and T3 sparsely white setose; S1–7 brown; S1 asetose, S2–3 sparsely yellow setose.

Female genitalia: densely arranged anteriorly directed setae present on T7–8 and S7–8; T8 with broad anterior rectangular apodeme; T9 formed by wide, rectangular sclerite with median protuberance; T9+10 entirely fused, T10 divided into 2 heavily sclerotised acanthophorite plates, 7–8 acanthophorite spurs per plate; 2 spermathecae, all equally large, formed by ± expanded weakly sclerotised ducts; individual spermathecal ducts short; S9 (furca) formed by 1 sclerite, ring-like (joined anteriorly and posteriorly), anterior furcal apodeme present, 2 lateral projections forming divided apodeme, lateral furcal apodeme absent, median furcal bridge absent.

###### Material examined:

**Mozambique:** Inhambane: 2 ♀ 1 ♂ Inhambane, 23°51'54"S; 035°22'60"E, -.-.-, Peters (♂ lectotype, ♀ paralectotypes, ZMHB); Sofala: 1 ♀ 3 ♂ 1? Beira, 19°50'37"S; 034°50'20"E, 2.ix.1961, G. Heinrich (AAM-000185–AAM-000189, CAS, CNC); 1 ♀ 1 ♂ Beira, 10.vii.1973, R. Erasmus (AAM-000287–AAM-000288, NMSA); 1 ♂ Beira, -.viii.1903, P. Krantz (AAM-000549, TMSA); 1 ♂ Beira, -.vi.1932, J. Ogilvie (AAM-000713, BMNH); 1 ♀ Upper Nhamapaza river, forest S Maringue, 17°57'52"S; 034°23'26"E, 11.vi.1929, P. Lesne (AAM-003272, MNHN); 1 ♀ 2 ♂ Nova Chupanga, 17°07'32"S; 034°51'34"E, 27.x.-, J. Surcouf (AAM-003268–AAM-003270, MNHN); Zambezia: 1 ♀ near Sone, 16°46'17"S; 037°45'16"E, 24.v.1928, P. Lesne (AAM-003271, MNHN).

###### Distribution:

Mozambique (Fig. 47). Biodiversity hotspot/high-biodiversity wilderness area: Coastal Forest of Eastern Africa/-.

###### Remarks:

In order to preserve taxonomic stability and make more universal the use of this specific name, the ♂ specimen from the syntype series deposited in the ZMHB is here designated as the lectotype, making the two remaining ♀ specimens paralectotypes. The specimen from Beira deposited in the TMSA (AAM-000549) has been studied by Hesse (1969: 277) and was identified as Syllegomydas arnoldi. I believe that it is a representative of the smaller species Syllegomydas dispar as this species appears to inhabit the coastal and lowland parts of Mozambique while the larger species Syllegomydas arnoldi appears to inhabit higher elevation areas within Zimbabwe (although 2 specimens from the Lowveld of South Africa from Kruger National Park are deposited in the SANC (South African National Collection of Insects, Pretoria, AAM-000088–AAM-000089)).

##### Syllegomydas (Notobates) heothinos sp. n.

urn:lsid:zoobank.org:act:C970D338-728B-4F9A-8B68-232C5BEEF33F

Figs 16–18, 20 40–41 45 48 

###### Etymology:

*heothinos* Greek adjective = eastern. Referring to the distribution in eastern Africa.

###### Diagnosis:

The species is distinguished from congeners by the relatively large size, the brown and yellow abdominal tergites in males and even more pronounced in females, the absence of lateral furcal apodemes in females, and its apparent distribution in Kenya and Uganda.

###### Description Male:

Head: black, facial gibbosity light brown, in general grey pruinose; width distinctly greater than thorax, interocular distance on vertex larger than at ventral eye margin, vertex between compound eyes ± horizontally straight, medially only slightly below dorsal eye margin, parafacial area less than ½ the width of central facial gibbosity; facial gibbosity distinct, well-developed and discernible in lateral view; mystax white, covering entire facial gibbosity; frons medially apruinose, laterally grey pruinose, vertex predominantly apruinose, only lateral margins grey pruinose, postgenae apruinose; setation: vertex white, frons white, ocp setae white, pocl setae white; ocellar triangle apruinose; proboscis light brown, short, about ½ length of oral cavity; labella small, as wide as prementum, as long as prementum, unsclerotised laterally; maxillary palpi cylindrical, light brown, longer than ½ the length of proboscis.

Antenna: brown or orange, scape and pedicel white setose dorsally and ventrally; postpedicel cylindrical in proximal ⅔, symmetrically bulbous in distal ⅓, ≥ 7.0 times as long as combined length of scape and pedicel; apical ‚seta-like‘ sensory element situated apically in cavity on postpedicel.

Thorax: dark brown to bluish-black, predominantly grey pruinose; scutum medially bluish-black, laterally brown, surface entirely smooth, predominantly grey pruinose, broad sublateral stripes (interrupted postsuturally) and narrow paramedial stripes (not reaching posterior margin) apruinose, scutal setation comprised of distinct rows of long dorsocentral setae and lateral scutal setae; dc setae pre- and postsuturally white, acr setae present, lateral scutal setae white, npl, spal, and pal setae absent; postpronotal lobe light brown, grey pruinose; proepisternum, lateral postpronotum, and postpronotal lobes long white setose; scutellum silver pruinose proximally, apruinose distally, asetose, apical scutellar setae absent; mesopostnotum, anatergite, and katatergite grey pruinose, mesopostnotum entirely long white setose, anatergite long white setose, katatergite long white setose; katatergite ± flat; anterior anepisternum asetose, supero-posterior anepisternum long white setose; posterior anepimeron long white setose, katepimeron white setose; metepimeron ± flat, yellow, grey pruinose, long white setose; metepisternum grey pruinose, asetose.

Leg: brown or light brown, setation predominantly white; all coxae grey pruinose, white setose; met trochanter setose medially; femora brown or light brown, met femora ± cylindrical only slightly wider than pro and mes femora, in distal ½ macrosetose, 1 antero-ventral and 1 postero-ventral row of macrosetae; all tibiae laterally arched, met tibia cylindrical, ventral keel absent; pro and mes tarsomere 1 about as long as individual tarsomeres 2, 3, or 4, met tarsomere 1 as long as combined length of tarsomeres 2–3; pulvilli well-developed, as long as well-developed claws, and as wide as base of claws; empodium absent.

Wing: length = (10.5–)12.0–12.5 mm; hyaline throughout, slightly brown stained along veins, veins brown, microtrichia absent; cells r_1_, r_4_, r_5_, m_3_, + cu*p* closed; C terminates at junction with R_1_; R_4_ terminates in R_1_; R_5_ terminates in R_1_; stump vein (R_3_) at base of R_4_ present, short not reaching R2; R_4_ and R_5_ widest apart medially; r-m distinct, R_4_+5 and M_1_ apart, connected by crossvein; M_1_ straight at r-m (not curving anteriorly), M_1_ (or M_1_+M_2_) terminates in R_1_; CuA_1_ and CuA_2_ split proximally to m-cu (cell m_3_ narrow proximally); m_3_+CuA_1_ do not terminate together in C; A_1_ undulating, cell A_1_ wide, A_1_ and wing margin further apart proximally than distally, alula well-developed; halter light brown.

Abdomen: brown and yellow; setation comprised of scattered white and brown setae, surface entirely smooth; T1 brown, narrow yellow posterior margin, T2–7 brown, broad yellow posterior margins, expanding antero-laterally particularly on T2–3; T1 and anterior ½ of T2 long white setose, remaining T2 and T3 brown setose; T predominantly apruinose; S1–7 brown; S1 asetose, S2 long white setose, S3 short brown setose; S predominantly apruinose; T2–4 parallel-sided and not constricted waist-like; bullae on T2 brown, oval, surface entirely smooth, T2 surface anterior to bullae smooth.

Male terminalia: T1–7 well-developed, entirely sclerotised, T8 postero-medially weakly sclerotised, with anterior transverse sclerotised bridge connecting lateral sclerites; T7–8 anteriorly with 2 lateral apodemes; S6 regular, without any special setation postero-medially, S8 well-developed and simple, not fused to T8 dorso-laterally, entire (undivided) ventro-medially; epandrium formed by single sclerite (fused medially ± entirely), pointed postero-laterally; subepandrial sclerite without lateral or median protuberances; hypandrium strongly concave, cup-shaped, entirely sclerotised ventrally (forming a single sclerite), entirely fused with gonocoxite, forming a gonocoxite-hypandrial complex; gonocoxites dorso-ventrally flattened in distal ½, higher in proximal ½, without median or lateral protuberance, gonocoxal apodeme absent; 2 functional aedeagal prongs, short and wide, medio-distally free, parallel or diverging laterally, distally straight or only diverging slightly laterally; aedeagal epimere present, distally simple, evenly rounded; lateral ejaculatory processes absent; ejaculatory apodeme formed by single dorso-ventrally oriented plate; ventro-median margin of dorsal aedeagal sheath heavily sclerotised (appearing entirely closed); dorsal aedeagal sheath long, sperm sac entirely covered; sperm sac appearing ± heavily sclerotised.

###### Description Female:

Head: brown, facial gibbosity light brown; parafacial area about as wide as ½ the width of central facial gibbosity; vertex medially apruinose, laterally grey pruinose.

Antenna: postpedicel ≥ 5.0 times as long as combined length of scape and pedicel.

Thorax: scutum brown, postero-medially bluish-black, scutal setation comprised of distinct rows of short dorsocentral setae and lateral scutal setae; proepisternum, lateral postpronotum, and postpronotal lobes short white setose; scutellum grey pruinose proximally, apruinose distally, asetose; supero-posterior anepisternum short white setose; metepimeron light brown.

Leg: all coxae apruinose, short white setose; met tarsomere 1 as long as combined length of tarsomeres 2–4.

Wing: length = 14.3–16.3 mm; slightly brown stained, darker brown around veins.

Abdomen: T1–7 yellow, narrow brown anterior margins that are widest laterally and medially; T1 and anterior ¼ of T2 long white setose, remaining T2 and T3 sparsely white setose; S1–7 brown, yellow postero-laterally; S1 asetose, S2–3 sparsely brown setose.

Female genitalia: densely arranged anteriorly directed setae present on T7–8 and S7–8; T8 with broad anterior rectangular apodeme; T9 formed by wide, rectangular sclerite with median protuberance; T9+10 entirely fused, T10 divided into 2 heavily sclerotised acanthophorite plates, 9–10 acanthophorite spurs per plate; 2 spermathecae, all equally large, formed by ± expanded heavily sclerotised ducts; individual spermathecal ducts short; S9 (furca) formed by 1 sclerite, ring-like (joined anteriorly and posteriorly), anterior furcal apodeme present, 2 lateral projections forming divided apodeme, lateral furcal apodeme absent, median furcal bridge absent.

###### Material examined:

**Kenya:** Eastern Province: 1 ♂ Archers Post, 00°39'00"N; 037°41'00"E, 5.v.1976, I. Bampton (AAM-001137 paratype, NMSA); 3 ♂ Athi River, 02°38'31"S; 038°21'59"E, 5–10.vii.1999, R. Copeland (AAM-001126 1 ♂ holotype, AAM-001127–AAM-001128 paratypes, NMKE); 1 ♂ Athi River, 2–9.viii.1999, R. Copeland (AAM-001129 paratype, NMKE); 2 ♂ Athi River, 24–27.v.1999, R. Copeland (AAM-001130–AAM-001131 paratypes, NMKE); 1 ♂ Athi River, 19–24.vii.1999, R. Copeland (AAM-001132 paratype, NMKE); 1 ♂ Lake Baringo, 00°37'05"N; 036°01'35"E, -.xii.1994, R. Copeland (AAM-001133 paratype, NMKE); 1 ♂ Samburu National Park, near Eawso Ng‘iro River, 00°34'05"N; 037°32'08"E, 874 m, Malaise Trap, riverine forest next to headquarters, 29.v.–12.vi.2007, R. Copeland (AAM-001134 paratype, NMKE); 2 ♂ Samburu National Park, near Eawso Ng‘iro River, 874 m, Malaise Trap, riverine forest next to headquarters, 12–16.vi.2007, R. Copeland (AAM-001135–AAM-001136 paratypes, NMKE); 2 ♀ Samburu National Park, near Eawso Ng‘iro River, 874 m, 26.vi.–10.vii.2007, R. Copeland (coll. Copeland); 1 ♀ 1 ♂ Kiboko, 02°10'00"S; 037°43'00"E, 16.vii.1981, R. Markham (AAM-000138–AAM-000139 paratypes, BMNH); 1 ♀ 5 ♂ Kiangeni River, Simba, 02°08'46"S; 037°36'28"E, -.vii.1958, B. Pearsons (AAM-001101–AAM-001106 paratypes, BMNH); 1 ♂ Makueni, 01°48'00"S; 037°37'00"E, -.-.1957, B. Pearsons (AAM-001108 paratype, BMNH); 1 ♀ British East Africa, -.-.-, S. Neave (AAM-001107 paratype, BMNH); **Uganda:** Karamoja: 1 ♂ Atumatak Catchment Area, 02°14'00"N; 034°38'00"E, in dry + sandy flume bed, -.xi.1960 (AAM-001109 paratype, BMNH).

###### Type locality and distribution:

Athi River (02°38'31"S; 038°21'59"E), Kenya. Kenya, Uganda (Fig. 48). Biodiversity hotspot/high-biodiversity wilderness area: -/-.

###### Remarks:

This species was first recognised as new by me based on material made available by R. Copeland in 2002. However, when I visited the BMNH in February 2010, I found several specimens labelled as types of a yet unpublished name by J. Bowden. Bowden had divided the species into three subspecies, the nominal subspecies based on the material from the Kiangani River and Makueni, one subspecies based on the single female from ‘British East Africa’, and another subspecies based on the single male from Uganda. The material from Kiboko collected in 1981 was interestingly not included by J. Bowden in his type material. I regard all these specimens as well as all other listed material as belonging to the same species. This species represents the first Mydidae known from Uganda (Fig. 48) and also the northernmost distribution of the subgenus Notobates.

**Figure 46.**
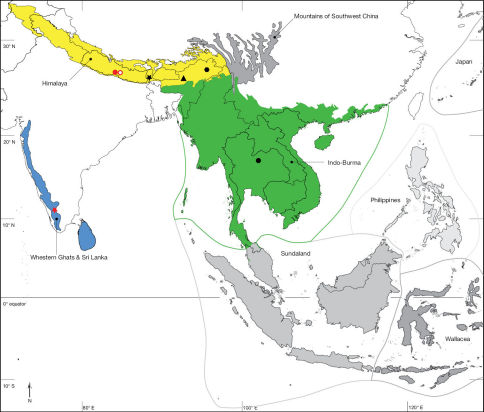
Map of the Oriental Region with biodiversity hotspots showing distribution of eastern species of Leptomydas. Leptomydas annulata comb. n. (black triangle), Leptomydas bucciferus comb. n. (black star), Leptomydas indianus (black pentagon), Leptomydas notos sp. n.(red star), Leptomydas rapti sp. n.(red circles, open circle = type locality), and Leptomydas tigris sp. n. (black circle).

**Figure 47.**
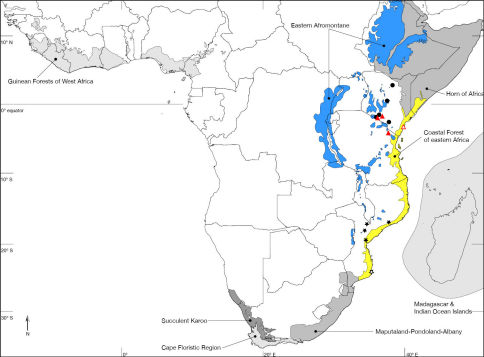
Map of the Afrotropical Region with biodiversity hotspots showing distribution of Mydaselpis ngurumani sp. n. (red triangle), Syllegomydas dispar (black star), Syllegomydas astrictus sp. n.(black circle). Type localities with open symbols.

**Figure 48.**
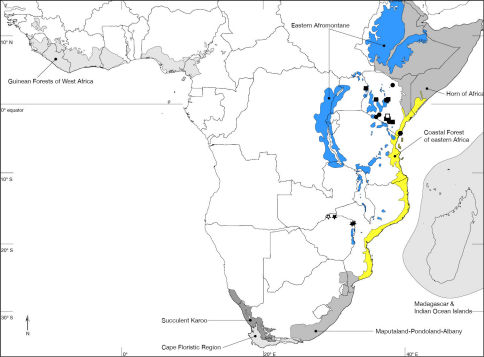
Map of the Afrotropical Region with biodiversity hotspots showing distribution of Syllegomydas elachys sp. n.(star), Syllegomydas heothinos sp. n.(square), and Vespiodes phaios sp. n. (pentagon). Type localities with open symbols.

## Discussion

The present paper provides descriptions of new species from areas from which Mydidae were not known before and therefore gives us a much better picture of the actual distribution of these interesting but rare flies world-wide. Eastern Africa, which was previously an empty spot on the map for Mydidae, actually harbours an interesting diversity of Mydidae species. Southern Africa has by far the greatest Mydidae diversity world-wide, but new collecting expeditions to western Africa will surely discover new species. The distribution of Leptomydinae within the Oriental Region (Fig. 46 and see below) has been expanded and the occurrence gap between the south-eastern Palaearctic Region (Afghanistan and Iran) and the eastern Oriental Region (Taiwan and the Yaeyama Islands of Japan) has been closed. For regularly updated distribution maps for all Mydidae species based on specimen occurrence data see http://www.mydidae.tdvia.de/mydidae_specimen_map.

### Oriental species of Leptomydas

The generic placement of the three new species of Leptomydas from the Oriental Region is not without question. Nagatomi and Tawaki (1985) reported for the first time Nemomydas Curran, 1934 from the north-eastern Oriental Region placing in it Nemomydas gruenbergi Hermann, 1914 known from Taiwan and the Yaeyama Islands of Japan. Nemomydas is otherwise only known from the Nearctic and northern Neotropical regions from south-western Canada in the north to Panama in the south (Hardy 1950, Steyskal 1956, Kondratieff and Welch 1990, Welch and Kondratieff 1991, 1994). Leptomydas on the other hand is widely distributed in the southern Palaearctic Region and in north-eastern India in the Oriental Region (Bequaert 1961, Richter 1997).

Curran’s (1934) original description of Nemomydas and also the added generic characters by Wilcox and Papavero (1971) do also apply to Leptomydas species studied by me, i.e., the type species Leptomydas lusitanicus (Wiedemann, 1820) from Spain, Leptomydas sardous (Costa, 1884) from Italy, and Leptomydas turcicus Bowden, 1983 from Turkey. The only morphological differences I can find among Old World and New World species, are male terminalia characters (particularly the development of median gonocoxal appendages). These small morphological differences of the male terminalia should, in my view, not be used to delineate separate genera. I therefore place the three new species in Leptomydas, which is the older name and exhibits a more-or-less uninterrupted distribution from Portugal in the west to central Thailand in the east.

Three species, Cephalocera annulata Brunetti, 1912, Leptomydas indianus Brunetti, 1912, and Syllegomydas bucciferus Séguy, 1928, from north-eastern India have been listed as unplaced species by Papavero and Knutson (1975). Based on the descriptions and wing venation, all of these species can be placed in Leptomydinae and do not belong to Cacatuopyginae (see key above), which is the other subfamily taxon distributed in the Oriental Region. The type specimens of Cephalocera annulata and Leptomydas indianus are supposed to be deposited in NZSI (Zoological Survey of India, Calcutta, India), but were unavailable for study. The type specimens of Syllegomydas bucciferus are deposited in the MNHN and have been studied. Cephalocera annulata and Syllegomydas bucciferus can therefore also be newly combined with Leptomydas.

**Leptomydas annulata (Brunetti, 1912), comb. n.**

**Leptomydas bucciferus (Séguy, 1928), comb. n.**

With the addition of these species, Leptomydas has now 17 valid species. Nemomydas gruenbergi might also need to be placed in Leptomydas, which will be tested with a phylogenetic study of Mydidae currently in preparation by the author.

### Mydaselpidini genera

The Mydaselpidini is a tribal taxon limited to the Afrotropical Region with currently three assigned genera, i.e., Afromydas Bequaert, 1961 (1 species), Mydaselpis (6 species), and Vespiodes (10 species) (Hesse 1969, Bowden 1980). Species of Mydaselpis, which are now known from southern Africa and Kenya, are morphologically very similar to Vespiodes, which is much more widely distributed in sub-Saharan Africa (see above). Afromydas guichardi Bequaert, 1961 is only known from the type locality in north-western Somalia. The three genera can at present only be distinguished by means of the male terminalia and in particular by the presence and shape of the aedeagal epimere that is absent in Mydaselpis, present and distally simple and evenly rounded in Vespiodes, and present and distally bifurcate in Afromydas. Hesse (1969: 31) described Vespiodes and distinguished it from Mydaselpis based on a more wasp-like and conopid-like appearance and the mentioned aedeagal characters and also highlighted that Afromydas might be a senior synonym of this genus (Hesse 1969: 41).

The monotypic genus Neolaparopsis Hesse, 1969, originally based on two female type specimens from Ndumo Game Reserve in north-eastern KwaZulu-Natal, South Africa, might need to be placed in Mydaselpidini as well. The morphological similarity of the females of Mydaselpis and Neolaparopsis was highlighted by Hesse (1969: 234), but he believed that Neolaparopsis is more similar to Arenomydas Hesse, 1969 and Nomoneura Bezzi, 1924, genera not placed by him in Mydaselpidini. Although Neolaparopsis puncturatus Hesse, 1969 does not show the wasp-like constriction of the abdomen that is present in several known Mydaselpis species (but not, for example, in a female specimen of Mydaselpis peringueyi Bezzi, 1924 in the SAMC) and Vespiodes, this constriction is also absent in females of Mydaselpis ngurumani sp. n. In addition, the female ovipositor possesses acanthophorite spurs as do females of Mydaselpis ngurumani sp. n., but as far as we know these spurs are reduced in other species of Mydaselpidini. Until recently, only female specimens of Neolaparopsis were known, but I received unidentified Mydidae specimens of both sexes for examination from J. Bosák (Olomouc, Czech Republic) that were collected in Tembe Elephant Park close to Ndumo Game Reserve and represent Neolaparopsis puncturatus (AAM-001143–AAM-001146). The male aedeagus possesses an aedeagal epimere that is laterally flattened and therefore of different shape than in nales of Afromydas and Vespiodes. Therefore, Neolaparopsis shows an interesting mixture of character states in regards to other Mydaselpidini genera. Future taxonomic research including a revision of the genus Vespiodes and phylogenetic analyses of the Mydaselpidini genera including Neolaparopsis, currently in preparation by the author, will shed light on the possible synonymy and placement of the discussed genera.

### Seasonal incidence

The following seasonality can be summarised from the studied material: Leptomydas notos sp. n.: May; Leptomydas rapti sp. n.: March–April; Leptomydas tigris sp. n.: February; Mydaselpis ngurumani sp. n.: January, June–July; Vespiodes phaios sp. n.: March; Syllegomydas astrictus sp. n.: June–August; Syllegomydas dispar: May–October; Syllegomydas elachys sp. n.: August–September; Syllegomydas heothinos sp. n.: May–August, November–December; Syllegomydas proximus: October–December. Mydaselpis ngurumani and Syllegomydas heothinos seem to exhibit two distinct flight periods each year whereas all other species have only been collected during a particular time of the year.

### Biodiversity hotspots and high-biodiversity wilderness areas

The biodiversity hotspots *sensu* Conservation International (Myers et al. 2000) are areas of high plant endemism in which the habitat has been destroyed to a considerable extant and which are under threat of more destruction. The high-biodiversity wilderness areas (Mittermeier et al. 2003) highlight those land masses that are largely undisturbed and have a very low population density. Evaluating the presence/absence of Diptera species in these priority areas earmarked for conservation can determine whether these species will also be preserved when funding is made available for their protection (e.g., Dikow et al. 2009). Of the 13 species dealt with in this contribution, of which 12 are mapped in Figs 46–48, 6 are endemic to one biodiversity hotspot. Vespiodes phaios sp. n. is endemic to the Coastal Forest of Eastern Africa hotspot and all species studied from the Oriental Region are endemic to a particular biodiversity hotspot, i.e., Leptomydas annulata comb. n., Leptomydas bucciferus comb. n., Leptomydas indianus, and Leptomydas rapti sp. n. are endemic to the Himalaya, Leptomydas tigris sp. n. to Indo-Burma, and Leptomydas notos sp. n. to the Western Ghats and Sri Lanka. Syllegomydas (Notobates) dispar occurs in the Coastal Forest of Eastern Africa hotspot, but also outside of it in lowland habitats of central Mozambique. Mydaselpis ngurumani sp. n. does occur in the Eastern Afromontane hotspot, but does primarily occur outside of this patchy biodiversity hotspot. While Syllegomydas elachys sp. n. is the only species that occurs in and is endemic to the high-biodiversity wilderness area Miombo-Mopane Woodland and Savannas, several known localities of Syllegomydas dispar do also occur within the boundaries of this vast high-biodiversity wilderness area. Syllegomydas astrictus sp. n., distributed throughout Kenya, and Syllegomydas proximus, distributed on the southern edge of the Sahara, do not occur in any biodiversity hotspot or high-biodiversity wilderness area *sensu* Conservation International.

## Supplementary Material

XML Treatment for Leptomydas notos

XML Treatment for Leptomydas rapti

XML Treatment for Leptomydas tigris

XML Treatment for Mydaselpis

XML Treatment for Mydaselpis ngurumani

XML Treatment for Vespiodes

XML Treatment for Vespiodes phaios

XML Treatment for Syllegomydas

XML Treatment for Syllegomydas (Syllegomydas) astrictus

XML Treatment for Syllegomydas (Syllegomydas) elachys

XML Treatment for Syllegomydas (Syllegomydas) proximus

XML Treatment for Syllegomydas (Notobates)

XML Treatment for Syllegomydas (Notobates) dispar

XML Treatment for Syllegomydas (Notobates) heothinos
